# Organophosphate exposures during pregnancy and child neurodevelopment: Recommendations for essential policy reforms

**DOI:** 10.1371/journal.pmed.1002671

**Published:** 2018-10-24

**Authors:** Irva Hertz-Picciotto, Jennifer B. Sass, Stephanie Engel, Deborah H. Bennett, Asa Bradman, Brenda Eskenazi, Bruce Lanphear, Robin Whyatt

**Affiliations:** 1 Environmental Health Sciences Center and Department of Public Health Sciences, School of Medicine, University of California Davis, Davis, California, United States of America; 2 Natural Resources Defense Council, Washington, DC, United States of America; 3 George Washington University, Washington, DC, United States of America; 4 Department of Epidemiology, University of North Carolina Chapel Hill, Chapel Hill, North Carolina, United States of America; 5 School of Public Health, University of California Berkeley, Berkeley, California, United States of America; 6 BC Children’s Hospital, Faculty of Health Sciences, Simon Fraser University, Vancouver, British Columbia, Canada; 7 Mailman School of Public Health and Children’s Center for Environmental Health at Columbia University, New York, New York, United States of America

## Abstract

In a Policy Forum, Irva Hertz-Picciotto and colleagues review the scientific evidence linking organophosphate pesticides to cognitive, behavioral, and neurological deficits in children and recommend actions to reduce exposures.

**Summary points**

- Widespread use of organophosphate (OP) pesticides to control insects has resulted in ubiquitous human exposures.
- High exposures to OP pesticides are responsible for poisonings and deaths, particularly in developing countries.
- Compelling evidence indicates that prenatal exposure at low levels is putting children at risk for cognitive and behavioral deficits and for neurodevelopmental disorders.

To protect children worldwide, we recommend the following:

- Governments phase out chlorpyrifos and other OP pesticides, monitor watersheds and other sources of human exposures, promote use of integrated pest management (IPM) through incentives and training in agroecology, and implement mandatory surveillance of pesticide-related illness.
- Health professions implement curricula on the hazards from OP pesticides in nursing and medical schools and in continuing medical education courses and educate their patients and the public about these hazards.
- Agricultural entities accelerate the development of nontoxic approaches to pest control through IPM and ensure the safety of workers through training and provision of protective equipment when toxic chemicals are to be used.

## Introduction

Organophosphate (OP) compounds were originally developed as human nerve gas agents during the 1930s–1940s, and some were later adapted as insecticides at lower doses [1]. High exposure to OP compounds leads to acute poisoning from the irreversible inhibition of the enzyme acetylcholinesterase (AChE), resulting in cholinergic syndrome (including narrowed pupils, excessive salivation, bronchoconstriction, mental confusion, convulsions or tremors, and in some cases, death). Additionally, delayed polyneuropathy has been described in association with high exposures [1].

In the United States, many OP pesticides—including malathion, dichlorvos, azinphos-methyl, and chlorpyrifos—were licensed for insecticidal use before requirements to evaluate human toxicity or ecologic effects were established [2]. Because OP pesticides rapidly degrade in the environment, they were considered safer than persistent organochlorine insecticides like DDT, aldrin, and dieldrin, but over 40 OP pesticides, including the most commonly used ones, are now considered by the US Environmental Protection Agency (EPA) [3] and/or the WHO Food and Agriculture Organization [4] to be moderately or highly hazardous to human health.

**Fig 1:**
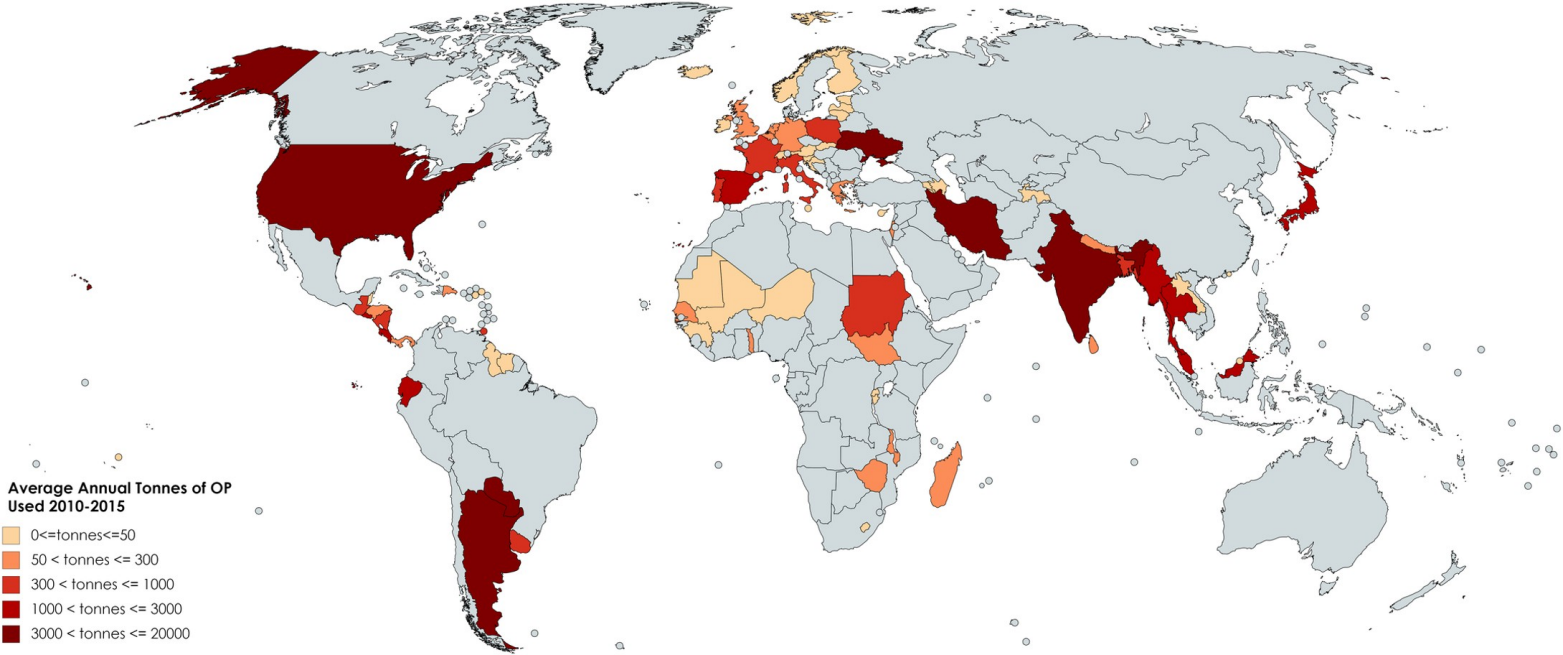
Average annual tonnes of OP pesticides used in agriculture, by country, 2010–2015. Darker shading indicates greater usage. Gray shading indicates that no data were available during that time period. For countries with data available for some but not all years during 2010–2015, the available data within that period were used. Source for US data was [6]; and for all other countries, [5]. *Map created with mapchart*.*net*. OP, organophosphate.

The most comprehensive global database on recent pesticide use includes information reported by 71 countries in five regions [5]. Annual use during 2010–2015 of OP pesticides in agriculture averages 1,145 tonnes (i.e., metric tons) for 13 African countries, 4,342 tonnes for 11 Caribbean and Central American countries, 10,013 tonnes for 24 European countries, 13,404 tonnes for 6 South American countries, and 29,554 tonnes for 17 Asian countries, with India dominating use. We additionally obtained data from the US [6] and have mapped total annual agricultural OP use by country (Fig 1) and total annual agricultural use by country per 1,000 square km (S1 Fig). Widespread use of OP pesticides in agriculture—as well as in homes, parks, schools, and hospitals and on golf courses, right-of-ways, and other public spaces—has led to ubiquitous human exposure.

OP pesticides present a range of health hazards. Here we review the scientific evidence of OP impacts on child neurodevelopment. In addition, we discuss inadequacies in current OP pesticide regulations and present recommendations for urgently needed policy change.

## Neurodevelopmental effects of OP pesticides

Systematic reviews and multiple epidemiologic studies in the US and other countries, spanning diverse populations in both urban and agricultural settings, have linked OP exposures during fetal development with poorer cognitive, behavioral, and social development in children [7–11]. Generally, levels of exposure in these studies are too low to induce measurable depression of cholinesterase in adults. In one review, adverse effects of OP pesticide exposure on neurodevelopment were seen in all but one of the 27 studies evaluated; the strongest associations occurred following prenatal exposures [9]. Outcomes associated with OP pesticide exposure to the fetus include abnormal primitive reflexes in newborns; mental and motor delays among preschoolers; and decreases in working and visual memory, processing speed, verbal comprehension, perceptual reasoning, and IQ among elementary school–age children. Prenatal exposures also elevated risks for symptoms or diagnoses of attention-deficit/hyperactivity disorder (ADHD) and autism spectrum disorder (ASD).

Consistent with the wide range of outcomes reported in human studies, the toxicity of early-life OP pesticides on neurodevelopmental end points has been confirmed in experimental animal studies. Parallel with epidemiologic findings, effects on cognition, motor activity, and social behaviors were repeatedly demonstrated in rodents dosed in early life with concentrations of OPs eliciting little to no inhibition of AChE in the brain [10,12]. The timing of exposure played a critical role in the biochemical and anatomic targets affected, as well as in the specific behavioral and developmental alterations evoked [12].

Since publication of the epidemiologic reviews, a higher likelihood of an ASD diagnosis was observed for children born to women residing within (versus beyond) 1.5 km of OP pesticide applications on agricultural fields; the strongest associations were for chlorpyrifos [13]. Another recent study showed that higher OP pesticide metabolite concentrations in maternal urine during pregnancy were associated with ASD traits identified in adolescence [14]. Other research teams reported residential proximity to agricultural OP use during fetal development to be associated with reduction in child’s IQ at age 7 years [15] and higher umbilical cord blood concentrations of chlorpyrifos with mild to moderate arm tremors in children at approximately age 11 years [16]. Risks for impaired neurodevelopment were greater among children of farmworkers, who experience higher exposures [17], and children with genetic susceptibility factors that reduce capacity to detoxify OP pesticides [7]. In the same study that examined ASD, moderate to severe developmental delay was associated with nearby applications of carbamates, similar to OP pesticides, but not with OP pesticides [13]. Two other studies, both conducted in urban cohorts of higher social and economic status, found no associations of OP pesticide metabolites with scores on intelligence tests [18,19]. Still, the weight of evidence clearly indicates that OP exposures during prenatal development are likely detrimental to brain function.

Accurate measurement of exposure is critical in environmental health studies. The OP pesticide studies determined exposure in various ways, ranging from quantification of OP metabolites in maternal urine collected during pregnancy and direct measurement of chlorpyrifos in umbilical cord blood to quantifying nearby pesticide use by geographically linking residential addresses with California’s database of commercial pesticide applications [20,21]. The California Pesticide Use Report Database, which contains specific pesticide quantity and the date and location of each application, has been validated by two exposure assessment studies, which showed that the amount applied within a few days to a week correlates highly with measured ambient air concentrations in nearby locations [22,23]. In the vast majority of studies reviewed, objective measures (both biologic markers and validated application data) were generated according to scientifically established protocols and obtained independently of the child’s outcome.

## Concerns at both high and low OP exposures

Critical to understanding the influences on early child neurodevelopment is the distinction between acute effects after high-level exposures versus sequelae from chronic lower exposures. As noted above, by inhibiting the enzyme AChE, high-level OPs cause acute, in some cases fatal, effects in humans [2]. Indeed, internationally, pesticide poisonings take a heavy toll, estimated at 200,000 deaths per year [24], with approximately 99% occurring in developing countries [25]. About 110,000 pesticide self-poisoning deaths occur each year globally, which represents an average across the reporting countries of 13.7% of all suicides [26], with a wide range from 0.9% in low- and middle-income European countries to 48.3% for low- and middle-income countries of the Western Pacific region.

Large quantities of highly hazardous OP pesticides are imported into developing countries. For example, OP pesticides ranked fourth among 24 chemical groups of pesticides imported into Central American countries [27], for which the two OP pesticides imported in the greatest quantity (terbufos and methamidophos) have been targeted for phaseout by the Rotterdam Convention, an international trade agreement on hazardous chemicals aimed at protecting human health and the environment [4,28]. Pesticide poisoning affects agricultural workers who often receive little or no instruction on the use of hazardous substances, are not provided with personal protective equipment, and/or operate application equipment that is not properly maintained. Additionally, overuse, misuse, and accidents have led to deaths of schoolchildren, e.g., in India in 2013, China in 2014, and Bangladesh in 2015, from consumption of meals with high levels of OP pesticides [4,24,29,30].

As tragic as these acute poisonings are, an OP pesticide exposure in the absence of overt poisoning does not imply that neurologic damage has not occurred—for both children and adults [31]). The US EPA concluded in 2016 that the existing epidemiologic literature provided “sufficient evidence that there are neurodevelopmental effects occurring at chlorpyrifos exposure levels below that required to cause acetylcholinesterase inhibition” [11]. Such chronic, low-level exposures are often overlooked or dismissed as benign because neither the pregnant woman nor the fetus shows clinically visible signs or symptoms. Furthermore, the developmental deficits do not manifest until months or years later. Indeed, the scientific consensus is that AChE inhibition is uninformative with regard to neurodevelopmental effects in children and that the toxic effects from chronic, low-level exposure occur at concentrations too low to inhibit cholinesterase [1,9]. The evidence thus indicates that OP pesticides can interfere with brain development at levels previously thought to be safe or inconsequential.

Hence, AChE inhibition cannot be used as a biomarker to identify neurodevelopmentally harmful OP pesticide exposures. Reliance on AChE inhibition for regulatory purposes obscures the serious threat that OP pesticides pose to early brain development and represents an unscientific and inadequate approach to health risk assessment. In fact, other effects appear likely to mediate the OP toxicity to neuronal systems that is foundational for childhood behavioral and cognitive deficits. Toxicologic evidence implicates OP pesticides in neuroinflammation, protein-kinase C receptor signaling, insulin resistance, dopaminergic and glutamatergic neurotransmission, and interference with DNA synthesis and nuclear transcription factor functioning, mechanisms highly relevant for brain development [12,32–34].

Indeed, as-yet-undiscovered harm may emerge from further follow-up of those exposed in early life. Outcomes from fetal exposures appear to be persistent, with associations observed into mid- and late childhood. One cohort repeatedly showed deficits in memory, IQ, and attention deficits or ADHD at ages 2, 3, 5, and 7 years, whereas another exhibited deficits in mental development and reasoning in infancy and at ages 6–9 years (reviewed in [8]). Children with high versus low chlorpyrifos concentrations in their umbilical cord blood had differences in brain volume in regions responsible for attention, receptive language processing, social cognition, and regulation of inhibition [35]. These neuroanatomic alterations, which potentially constitute a pathway from pesticide exposure to the associated behavioral and cognitive deficits, may be permanent.

## Pesticide regulation

Pesticide regulations vary widely across the globe. As with pesticide usage, no database has consolidated this information for all countries. Table 1 shows available data on 47 OP insecticides [36] banned by one or more countries, as well as the level of health hazard and the number of countries that have banned each OP pesticide. The most comprehensive database available on current governmental regulation of pesticides provides data covering 39 of these 47 OP insecticides, obtained from 106 countries outside the US [37]. Included in this database are total bans, along with denials of approval, but not restrictions. Of the 106 countries, 81% have regulated one or more of the 39 OP insecticides [37]. The 28 countries of the European Union have taken action on the most OP pesticides (33). Additional countries that have banned more than 10 include the US (26), Cambodia (15), China (15), Saudi Arabia (15), Guinea (12), Korea (12), Mauritania (12), and Thailand (12). Notably, having regulations in place does not necessarily mean that they are enforced. Furthermore, some of the most toxic OP pesticides that are banned across dozens of countries are exported elsewhere, often to developing countries and sometimes in large quantities, for example, to Costa Rica and Guatemala [27]. In Mexico, at least a dozen OP pesticides that are classified as highly hazardous by the WHO Food and Agriculture Organization are used [38].

**Table 1:** OP insecticides, hazard levels, and number of countries banning them.

	Compound^1^	Hazard level			Number of countries (outside US) that have banned it^2^	Banned OPs in US designated by X. All other OPs on list are currently registered for use in the US^3^
		US EPA^4^	FAO-WHO^5^	PAN^2^		
1	Acephate	M	M	H	31	
2	Azinphos-methyl	H	H	H	39	X
3	Cadusafos	**	H	H	31	
4	Chlorethoxyphos	**	E	H	29	
5	Chlorfenvinphos	H	H	H	35	X
6	Chlorpyrifos	M	M	H	2	
7	Chlorpyrifos-methyl	**	S	H	1	
8	Chlorthiophos^6^^,^^7^	H	**	**	**	X
9	Coumaphos	H	H	H	30	
10	Dichlorfos (dichlorvos)	M	H	H	32	
11	Dialifor/dialifos^6^^,^^7^	H	**	**	**	X
12	Diazinon	M	M	H	30	
13	Dicrotophos	H	H	H	34	
14	Dimethoate	**	M	H	4	
15	Dioxathion^6^^,^^7^	H	**	**	**	X
16	Disulfoton	H	E	H	38	X
17	Ethion	M	M	—	30	X
18	Ethoprop (ethoprophos)	M	E	H	8	
19	Ethyl parathion^7^	H	**	**	**	X
20	Fenamiphos	H	H	H	6	X
21	Fenitrothion	M	M	H	28	
22	Fenthion	M	M	H	30	X
23	Fonofos (fenophos)^6^	H	**	—	33	X
24	Isazophos^6^^,^^7^	**	**	**	**	X
25	Isofenphos^6^	H	**	—	29	X
26	Malathion	M	S	H	2	
27	Methamidophos	H	H	H	49	X
28	Methidathion	H	H	H	34	X
29	Methyl parathion	H	E	H	59	X
30	Mevinphos	H	E	H	37	X
31	Monocrotophos	H	H	H	60	X
32	Naled	M	M	H	28	
33	Oxydemeton-methyl	M	H	H	30	X
34	Phorate	H	E	H	37	
35	Phosalone	M	M	—	29	X
36	Phosmet^7^	M	M	**	**	
37	Phosphamidon	H	E	H	49	X
38	Phostebupirim^7^	**	**	**	**	
39	Pirimiphos-methyl^7^	M	M	**	**	
40	Profenofos	M	M	H	29	X
41	Propetamphos	M	H	H	28	X
42	Sulfotepp	H	E	H	32	X
43	Sulprofos^6^^,^^7^	M	**	**	**	X
44	Temephos	M	S	H	28	X
45	Terbufos	H	E	H	34	
46	Tetrachlorvinphos	M	**	H	28	
47	Trichlorfon	M	M	H	32	

Level of hazard: E, extreme; H, high; M, moderate; S, slight

**, not classified;—, not H (PAN^6^ ranking); X, banned in the US.

^1^ This list of OP insecticides is taken from the US EPA Office of Pesticide Programs “Organophosphorus Cumulative Risk Assessment, 2006 Update” [36] (Table ES-1, p. 16 “OP Pesticides Considered in the 2006 Update of the Cumulative Risk Assessment”), from which we have excluded those pesticides that are not insecticides.

^2^ Hazard Ranking and number of countries that banned: from PAN International Consolidated List of Banned Pesticides [37] (http://pan-international.org/pan-international-consolidated-list-of-banned-pesticides/). Methods and sources for collection of these data are described in the Explanatory Notes: (http://pan-international.org/wp-content/uploads/Consolidated-List-of-Bans-Explanatory-2017April.pdf). This list does not include restrictions, only bans or decisions to not approve.

^3^ A "banned" pesticide in the US is defined as a pesticide for which all registered uses have been prohibited by final EPA action and includes pesticides that have been withdrawn through voluntary agreements between industry and the US EPA. Status of OPs that are either banned or registered for use in the US provided in personal communication from Yu-Ting Guilaran (Director, Pesticide Re-evaluation Division, Office of Pesticide Programs, US EPA) to JBS, July 12, 13, and 23, 2018.

^4^ Hazard ranking [3].

^5^ Hazard ranking [4]: The concept of and criteria for “Highly Hazardous Pesticides” was initially described in the JMPM second report in 2008,”Report of the 2nd FAO/WHO Joint Meeting on Pesticide Management” (last accessed July 2018) (http://www.fao.org/fileadmin/templates/agphome/documents/Pests_Pesticides/Code/Report.pdf). As scientific understanding of mechanisms for pesticide toxicity has advanced, these have been included, as described in the 2016 publication of “International Code of Conduct on Pesticide Management Guidelines on Highly Hazardous Pesticides” (http://apps.who.int/iris/bitstream/handle/10665/205561/9789241510417_eng.pdf;jsessionid=D3B3CCA5B28692A5F3D437B2CF7F0AA0?sequence=1). The FAO-WHO JMPM defined banned pesticides thus: “Banned pesticide means a pesticide all uses of which have been prohibited by final regulatory action, in order to protect human health or the environment. It includes a pesticide that has been refused approval for first-time use, or has been withdrawn by industry either from the domestic market or from further consideration in the domestic approval process, and where there is clear evidence that such action has been taken in order to protect human health or the environment.”

^6^ Considered to be obsolete or no longer used as a pesticide, according to the WHO Recommended Classification of Pesticide Hazards, 2010.

^7^ Not included in the PAN database.

Abbreviations: EPA, Environmental Protection Agency; FAO-WHO, WHO Food and Agriculture Organization; JMPM, Joint FAO/WHO Meeting on Pesticide Management; OP, organophosphate; PAN, Pesticide Action Network.

Within the US, the EPA regulates pesticides under two overlapping statutes—the Federal Food, Drug, and Cosmetic Act (FFDCA) and the Federal Insecticide, Fungicide, and Rodenticide Act (FIFRA). Many of the insecticides banned in the US were initially licensed prior to 1970, when required health and safety assessment was minimal and before the US EPA was formed. As a result of legislation in the 1970s requiring increased health and safety studies, voluntary agreements were reached between manufacturers and the EPA to cancel or phase out registrations for some pesticides, including 18 OP insecticides.

In 1996, the Food Quality Protection Act (FQPA) amended FIFRA and FFDCA by requiring the EPA to include additional safety factors to protect children because of their greater exposures and heightened susceptibility [39]. Children have larger body burdens of pesticides because of greater intake of food, water, and air than adults, per unit of their body weight; they explore the world through mouthing behaviors; and they frequently crawl or play on floors where pesticides and other toxic chemicals settle. Heightened susceptibility during early years arises in part from immature detoxifying enzyme systems, including paraoxonase 1 (PON1) [7,40,41]. Under the FQPA, the EPA must show that there is reasonable certainty that no harm will result from aggregate exposure to the pesticide, including all anticipated dietary exposures and all other exposures for which there is reliable information.

After passage of the FQPA, OP pesticide use across all market sectors declined by over 70%, from 70 million pounds per year (lbs/yr) in 2000 to about 20 million lbs/yr in 2012 (the most recent available data) [6]. By 2002, most nonagricultural uses were phased out by agreements between the EPA and the pesticide manufacturers, based on results of EPA risk assessments for chlorpyrifos and diazinon showing unacceptably high risks to residents, particularly children, from residential pest control [42,43]. The volume of OP pesticides used on foods commonly consumed by children, such as fruits, decreased by 57% between 1994 and 2004, from 28 to 12 million pounds (12,701 to 5,443 metric tonnes) of active ingredient applied annually [44]. This action resulted in dramatic reductions in blood and urine concentrations of OPs among the US population [45]. However, agricultural OP pesticide use continues to contribute to exposures for farmworkers, their families [15], and residents in homes, children in schools, and other bystanders near farmlands [23], as well as to food and drinking water contamination that affects a broader population.

In 2016, the EPA concluded that exposure to chlorpyrifos—the most commonly used OP insecticide in the US—from either food or drinking water alone could lead to unacceptably high population exposures and determined that some reproductive-aged women, infants, and children consumed levels of chlorpyrifos substantially above the acceptable level for these vulnerable life stages [11]. The EPA also identified numerous scenarios that could result in unsafe exposures for agricultural workers and bystanders. For these reasons, as required by law, the EPA proposed to revoke all standards (called tolerances) that permit residues of chlorpyrifos on food. Revocation of these tolerances would essentially ban this OP on food crops [11]. However, in March 2017, despite overwhelming evidence of harm and contrary to the EPA’s own risk assessments, the Trump administration EPA announced that “the science addressing neurodevelopmental effects remains unresolved, and that further evaluation of the science … [therefore] is warranted to achieve greater certainty as to whether the potential exists for adverse neurodevelopmental effects to occur from current human exposures to chlorpyrifos,” concluding that the EPA would not cancel any uses of chlorpyrifos [46]. This action would delay potential regulatory action until October 2022. However, on August 9, 2018, the US Court of Appeals for the Ninth Circuit ordered the EPA to finalize the ban on chlorpyrifos within 60 days, including a ban on all US sales and a prohibition of food contaminated by the insecticide from reaching the US market. The court based its decision on the EPA’s 2016 findings that the pesticide fails to meet federal safety standards and is particularly harmful to infants and children. In September 2018, the EPA filed a petition for a rehearing of the chlorpyrifos case.

## Recommendations

In 2014, the American Academy of Pediatrics called for pediatricians and governments to recognize and reduce pesticide exposures through education, pesticide labeling, public health surveillance, and regulatory action [47]. In 2016, an independent group of scientists and health professionals published the Project TENDR Consensus Statement as a national call to action to significantly reduce exposures to chemicals—including OP pesticides—that have been identified as putting children in the US, and likely throughout the world, at increased risk of neurodevelopmental disorders [48]. Project TENDR concluded that the evidence of significant risks to children’s neurodevelopment from OP pesticide exposure warrants strong regulatory action. In 2017, a United Nations report on the Right to Food called for changes to agricultural practices to ensure food that is safe, free from pesticides, and qualitatively adequate [24]. To achieve the goal of reducing exposures to OP insecticides, we therefore propose an action plan for governments, public health and medical institutions or organizations, and agricultural entities. Our recommendations are detailed in Box 1. These steps would markedly reduce prenatal and childhood exposures to OP pesticides.

**Box 1. Recommendations to move towards elimination of human exposures to OP pesticides**

We recommend the following actions by governments:

- National and state or provincial governments, globally: phase out use of all OPs in agriculture;
- National and state or provincial governments, globally: ban nonagricultural use of all OPs, including household products;
- US EPA: revoke all food tolerances for chlorpyrifos, as the agency previously proposed;
- US EPA and state governments: phase out the use of all other OPs in agriculture;
- US EPA: ban nonagricultural pest control uses of the few remaining OPs;
- In the interim, national, state, and local agencies: take steps to reduce human exposure (e.g., require advance notification to nearby residents and schools before applications of OP pesticides; implement restrictions on application methods such as aerial spraying and air blast to reduce drift exposures and to protect water and sensitive sites such as homes and schools);
- National, state, and local agencies: conduct regular monitoring of watersheds to ensure OPs do not continue to pollute lakes, rivers, and streams, including those that are sources of drinking water, and implement targeted monitoring of drinking water;
- National and state agencies: establish an effective comprehensive pesticide use and illness reporting program either nationally or through coordinated statewide programs.

We recommend that medical schools, public health programs, and healthcare associations:

- organize continuing medical education courses to educate healthcare providers on both acute and chronic effects of exposures to toxic chemicals, including how to recognize and treat children who received high OP exposures; how to advise pregnant women and parents of young children on steps they can take to avoid pesticide exposures from lice, flea, and tick treatments [49], lawn and garden products, and applications in nearby agricultural land, golf courses, schools, and shopping malls; and how properly to clean potential pesticide residues from fruits and vegetables and to identify which produce contain the highest levels;
- educate health providers on the necessary reporting of pesticide poisonings to state surveillance;
- encourage schools of nursing and medicine to incorporate curricula on environmental hazards that include pesticides and medical boards to include environmental health in their examinations.

We recommend that agricultural entities:

- provide enhanced training for workers, in the most appropriate languages and at relevant educational levels, on the handling and application of pesticides and on the worker protection standards. In the US, this means EPA Worker Protection Standards training at the required frequency;
- educate workers on how to avoid take-home exposures to their families;
- institute environmentally friendly approaches to control pests—integrated pest management (IPM)—with a goal to eliminate or minimize toxic chemicals in our food sources.

Exemplary actions at various governmental levels have been taken. At the multinational level the EU chose to not approve close to 200 pesticides, of which over 20 are OPs, and multiple individual countries have instituted bans on OPs such as dichlorvos, methamidophos, and methyl parathion [37]. In the US, California has taken steps to limit agricultural use of pesticides near schools and childcare facilities when children are present [50], and Hawaii recently banned the distribution, sale, transport, and use of any pesticide containing chlorpyrifos as an active ingredient [51].

In reducing OP pesticide usage, toxic effects from substitute or replacement chemicals require scrutiny. Pyrethroid pesticides have replaced OPs as the main class of insecticides in residential pest control products, but recent rodent laboratory studies and epidemiologic studies suggest that prenatal pyrethroid pesticide exposures may also increase the risk of adverse neurodevelopment and behaviors and negative emotions [13,52–54]. Neonicotinoid pesticides are now the fastest-growing class of insecticides used on crops in the US [55]; they are persistent in plants, soil, and water and highly toxic to invertebrates, including endangered aquatic species, bees, and other beneficial insects [56]. Moreover, the impacts of broad and systemic pesticide use are well documented to have had significant negative ecological consequences affecting terrestrial, aquatic, wetland, marine, and benthic habitats and posing risks to ecosystem functioning and resilience.

What are the alternatives, if synthetic pesticides other than OPs are also neurotoxic?

Agriculture represents the vast majority of OP pesticide use, which includes both crop and livestock production. Widespread implementation of IPM is needed to reduce this use. IPM is a reduced-risk pest management strategy that emphasizes inspection, monitoring, prevention, and pest control using the least toxic methods including (agri)cultural practices such as intercropping (growing two or more crops in close proximity, which can reduce susceptibility to disease and pests), crop rotation, and cover crops (to reduce soil erosion and improve soil health); physical controls such as traps or bug vacuums; habitat management that encourages beneficial insects; and biological control, such as the release of parasitic wasps to control aphids, with pesticides used only as a last resort. When used, least-toxic pesticides are chosen first, such as materials approved for organic farming (e.g., *Bacillus thuringiensis* to control Lepidoptera) [57].

While IPM strategies do not, in principle, forbid the use of OP and other neurotoxic pesticides, these higher-risk materials serve as a last resort and should be applied in a way that protects human and environmental health. That most crops produced with OP pesticides are also produced organically provides compelling evidence that OP pesticides are not essential [58]. Some recalcitrant pests may be difficult to manage with less toxic pesticides, which in some instances may result in lower yields or higher production costs, reducing competitiveness. Recent research, however, indicates that crop yields from organic and other alternative production systems are increasing and in some cases match conventional yields [59]; these approaches additionally would likely reduce external costs to public health and the environment [60]. To ensure that farmers are not threatened with rising costs and thinner profit margins, many agricultural trade and policy organizations recommend increased government support for extension research and outreach needed to support transitions to less toxic materials [61].

Public health, a second use of OP pesticides, represents a small fraction of their applications. For example, OP pesticides are used for mosquito and other vector control to prevent vector-borne diseases such as Zika virus or West Nile virus. We do not recommend abrupt changes in pest management that would increase the risk of exposure to these viruses. We do advocate increased funding for better understanding of the ecology and biology of these and other vectors and the diseases they spread and alternative methods to control them without the use of OP or other neurotoxic pesticides. The historical example of the Mediterranean fruit fly in California, a serious invasive agricultural pest, provides a model for application to disease vectors. In the early 1990s, state officials used helicopters to spray malathion over residential areas where over 2 million people resided [62]. Subsequent concerns [63] resulted in development of a comprehensive sterile fruit fly release program that, combined with spot treatments often using organically approved pesticides, has successfully controlled infestations without the need for OP pesticide applications over wide swaths of residential areas [64,65]. Similar strategies should also be considered for new invasive species, such as the spotted lanternfly, currently threatening eastern US ecosystems and agriculture. Integrated vector management would favor using least-toxic options.

Structural, indoor, and landscape pesticide applications, the third category of OP uses, can result in high exposures. Dichlorvos, an OP already banned in many countries, is still permitted indoors by the US government for flying insects. Similarly, malathion is still sold for landscape and garden use. Given risks of adverse health effects due to chronic, low-level exposures and reported acute poisoning of consumers in the US [66], we recommend that all remaining structural, indoor, and landscape use of OPs be phased out immediately, especially in environments where children are present. Basic IPM principles should be applied in these environments, including pest exclusion (i.e., screens) and traps.

To preserve health and sustainability, both indoor and outdoor pest management must ultimately rely on nontoxic or less toxic alternatives; simultaneously, agriculture needs stronger support to move towards a systems approach that minimizes use of neurotoxic pesticides while providing healthy food and economic sustainability for farmers. The Report on the Right to Food by the Special Rapporteur to the UN General Assembly articulates a similar philosophy: in order to successfully reduce or eliminate use of hazardous pesticides, the international community’s efforts will need to address the ecologic, social, and economic factors currently embedded in agricultural policies. At the national level, this will require challenging agrochemical-dependent farming to restructure and seek the safest feasible alternatives [24]. We join the American Academy of Pediatrics and the UN in recommending close surveillance of pesticide poisonings, incentives for nonchemical approaches to pest control, monitoring of water and food sources of pesticides, and enforcement of the public’s right to know through full disclosure, labeling, and further communications for pesticide formulations and for residues in food, water, and elsewhere. Finally, we believe it is an ethical and social responsibility for civil society, for the medical profession, and for the agricultural industry to disseminate widely to the general public what is known about the sources of pesticide exposures and their adverse impacts on health and to develop training programs in agroecology in order to achieve a paradigm shift in food production.

## Supporting information

S1 Fig Average annual tonnes of OP pesticides used in agriculture per 1,000 square km, by country, 2010–2015. Darker shading indicates greater usage per 1,000 square km. Gray shading indicates that no data were available during that time period. For countries with data available for some but not all years during 2010–2015, the available data within that period were used. Source for US data was [6]; and for all other countries, [5]. *Map created with mapchart*.*net*. OP, organophosphate.
(TIF)

S1 Text Spanish translation of full article. (DOCX)

S2 Text Chinese translation of full article. (DOCX)

S3 Text French translation of summary bullet points. (DOCX)

S4 Text Italian translation of summary bullet points. (DOCX)

## Abbreviations

AChE: acetylcholinesterase
ADHD: attention-deficit/hyperactivity disorder
ASD: autism spectrum disorder
EPA: Environmental Protection Agency
FFDCA: Federal Food, Drug, and Cosmetic Act
FIFRA: Federal Insecticide, Fungicide, and Rodenticide Act
FQPA: Food Quality Protection Act
IPM: integrated pest management
JMPM: Joint FAO/WHO Meeting on Pesticide Management
lbs/yr: pounds per year
OP: organophosphate
PAN: Pesticide Action Network
PON1: paraoxonase 1
