# Supplementary figures and images for: Organophosphate exposures during pregnancy and child neurodevelopment: Recommendations for essential policy reforms
Source: PLoS Med. 2018 Oct 24;15(10):e1002671. doi: 10.1371/journal.pmed.1002671 (PMC6200179; doi:10.1371/journal.pmed.1002671)

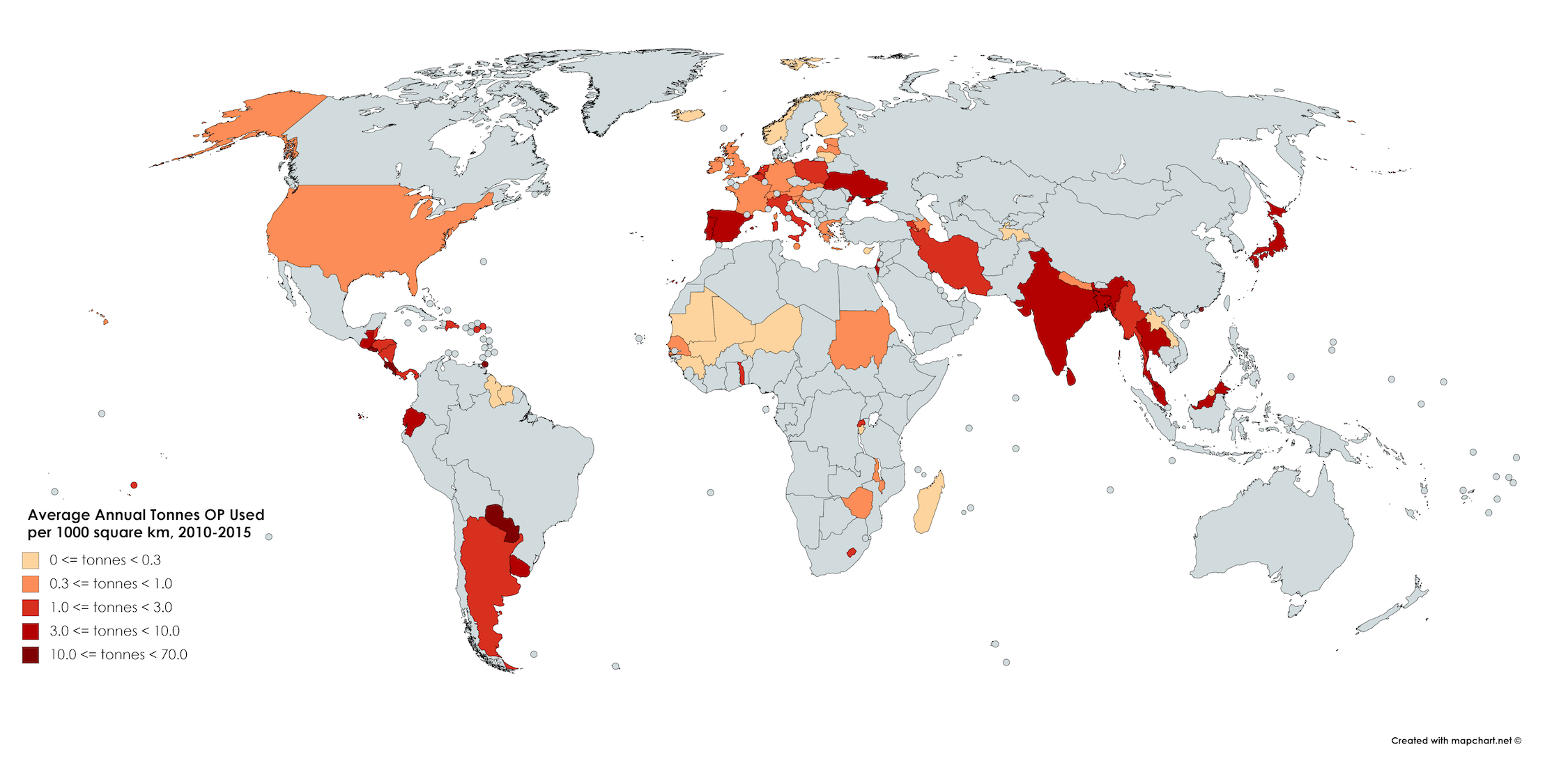

Supplement: S1 Fig — Darker shading indicates greater usage per 1,000 square km. Gray shading indicates that no data were available during that time period. For countries with data available for some but not all years during 2010–2015, the available data within that period were used. Source for US data was [6]; and for all other countries, [5]. Map created with mapchart.net. OP, organophosphate. (TIF) [file pmed.1002671.s001.tif]
