# Supplementary material for: Organophosphate exposures during pregnancy and child neurodevelopment: Recommendations for essential policy reforms
Source: PLoS Med. 2018 Oct 24;15(10):e1002671. doi: 10.1371/journal.pmed.1002671 (PMC6200179; doi:10.1371/journal.pmed.1002671)
Supplement: S1 Text — (DOCX) [file pmed.1002671.s002.docx]

**Exposición a organofosforados durante el embarazo y el desarrollo neurológico del niño: Recomendaciones para la reforma de las políticas esenciales**

Irva Hertz-Picciotto^1*^, Jennifer B. Sass^2^, Stephanie Engel^3^, Deborah H, Bennett^1^, Asa Bradman^4^, Brenda Eskenazi^4^, Bruce Lanphear^5^, Robin Whyatt^6^.

Afiliaciones:

1 Centro de Ciencias de la Salud Ambiental y Departamento de Ciencias de la Salud Pública, Facultad de Medicina, Universidad de California Davis, Davis, CA

2 Consejo de Defensa de Recursos Naturales y Universidad George Washington, Washington DC

3 Departamento de Epidemiología, Universidad de Carolina del Norte Chapel Hill, Chapel Hill, NC

4 Escuela de Salud Pública, Universidad de California Berkeley, Berkeley, CA

5 BC Hospital de Niños, Facultad de Ciencias de la Salud, Universidad Simon Fraser, Vancouver, BC, Canadá

6 Escuela de Salud Pública de Mailman y Centro de Salud Ambiental para Niños de la Universidad de Columbia, Nueva York, NY

* ihp@ucdavis.edu

Abreviaciones:

AChE, acetilcolinesterasa; cociente intelectual (CI); EPA, Agencia de Protección Ambiental de Estados Unidos; FFDCA, la Ley Federal de Alimentos, Medicamentos y Cosméticos; FIFRA, la Ley Federal de Insecticidas, Fungicidas y Rodenticidas; FQPA, la Ley de Protección de la Calidad de los Alimentos; MIP, manejo integrado de plagas; JMPM: Reunión conjunta FAO / OMS sobre manejo de plaguicidas; lbs/yr, libras por año; OF, organofosforado; PAN, Red de Acción de Plaguicidas; PON1, paraoxonasa ; TDAH, trastorno por déficit de atención e hiperactividad; TEA, trastorno del espectro autista

Título corto: Organofosforados y Neurodesarrollo: Revisión y Recomendaciones

**Resumen Ejecutivo**

- El uso generalizado de plaguicidas organofosforados (OF) para controlar los insectos ha dado lugar a exposiciones humanas ubicuas.
- La exposición elevada a los plaguicidas OFs son responsables de las intoxicaciones y muertes, especialmente en los países en desarrollo.
- La evidencia convincente indica que la exposición prenatal a niveles bajos está poniendo a los niños en riesgo de déficits cognitivos y conductuales.

Para proteger a los niños en todo el mundo, recomendamos que:

- Los gobiernos eliminen gradualmente clorpirifos y otros plaguicidas OF; monitoreen las cuencas hidrográficas y otras fuentes de exposición humana; promuevan el uso del manejo integrado de plagas (MIP) a través de incentivos y capacitación en agroecología; e implementen vigilancia obligatoria de enfermedades relacionadas con plaguicidas.
- Los profesionales de la salud implementen currículos sobre los peligros de los plaguicidas OF en las escuelas de enfermería y medicina y en cursos de educación continua en medicina; y eduquen a sus pacientes y al público acerca de estos peligros.
- Las entidades agrícolas aceleren el desarrollo de enfoque no tóxico para el control de plagas a través de MIP; y garanticen la seguridad de los trabajadores mediante la capacitación y el suministro de equipo de protección cuando se utilicen productos químicos tóxicos.

**Introducción**

Los compuestos organofosforados (OF) fueron originalmente desarrollados como agentes de gas nervioso humano durante los años 1930-1940, y algunos fueron más tarde adaptados como insecticidas en dosis más bajas [1]. La alta exposición a compuestos OF conduce a intoxicación aguda por la inhibición irreversible de la enzima acetilcolinesterasa, lo que resulta en el síndrome colinérgico (incluyendo pupilas estrechas, salivación excesiva, broncoconstricción, confusión mental, convulsiones o temblores, y en algunos casos muerte). Adicionalmente, polineuropatía retardada se ha descrito en asociación con altas exposiciones [1].

En los Estados Unidos, muchos plaguicidas OF - incluyendo malatión, diclorvos, azinfos-metil, y clorpirifos – estaban autorizados para uso insecticida antes de que se hayan hecho evaluaciones de toxicidad humana o se hayan comprobado los efectos ecológicos [2]. Porque plaguicidas OF rápidamente se degradan en el medio ambiente, fueron considerados más seguros que los insecticidas organoclorados persistentes como el DDT, aldrín, dieldrín, pero 40 plaguicidas OF, incluyendo los más comúnmente utilizados, son ahora considerados por la Agencia de Protección Ambiental de Estados Unidos (EPA) [3] y/o la Organización de las Naciones Unidas para la Alimentación y la Agricultura [4] de moderada o alta peligrosidad para la salud humana.

La base de datos mundial más completa sobre el uso reciente de plaguicidas incluye información reportada por 71 países en cinco regiones [5]. El uso anual durante 2010-2015 de plaguicidas OF en la agricultura promedia fue 1.145 toneladas (es decir, toneladas métricas) para 13 países africanos; 4.342 toneladas para 11 países del Caribe y Centroamérica; 10.013 toneladas para 24 países europeos; 13.404 toneladas para 6 países sudamericanos; y 29.554 toneladas para 17 países asiáticos con uso predominante en la India. Además, obtuvimos datos de los EE.UU. [6] e hicimos un mapa del uso agrícola anual de OF por país (Fig. 1) y el uso agrícola anual por país por cada 1.000 km cuadrados (Fig. 1- Suplementaria). El uso generalizado de plaguicidas OF en la agricultura, así como en hogares, parques, escuelas y hospitales; y en campos de golf, derechos de paso y otros espacios públicos, han llevado a la exposición humana ubicua.

Los plaguicidas OF presentan una gama de riesgos para la salud. Aquí revisaremos la evidencia científica de los impactos de plaguicidas OF en el neurodesarrollo infantil. Además, discutiremos las deficiencias en las regulaciones actuales de plaguicidas OF y presentaremos recomendaciones para el cambio necesario y urgente de políticas.

**Efectos de plaguicidas OF en el Neurodesarrollo**

Revisiones sistemáticas y varios estudios epidemiológicos en los EE.UU. y otros países, que abarcan diversas poblaciones, tanto en entornos urbanos y agrícolas, han relacionado exposiciones de OF durante el desarrollo fetal con un bajo desarrollo cognitivo, conductual y social en niños [7-11]. En general, los niveles de exposición en estos estudios son demasiado bajos para inducir una depresión medible de la colinesterasa en adultos. En una revisión, los efectos adversos de la exposición a plaguicidas OF en el neurodesarrollo se observaron en todos menos uno de los 27 estudios evaluados; las asociaciones más fuertes ocurrieron después de exposiciones prenatales [9]. Resultados asociados con la exposición de plaguicidas OF en el feto incluyen reflejos primitivos anormales en recién nacidos; retrasos mentales y motrices en edad pre-escolar; y decrementos en la memoria visual y de trabajo, velocidad de procesamiento, comprensión verbal, razonamiento perceptual y cociente or coeficiente intelectual (CI) entre niños de edad escolar primaria. Las exposiciones prenatales también aumentan los riesgos de síntomas o diagnósticosdel trastorno por déficit de atención e hiperactividad (TDAH) y el trastorno del espectro autista (TEA).

De acuerdo con la amplia gama de resultados reportados en estudios humanos, la toxicidad de los plaguicidas OF a una edad temprana, en los puntos finales del desarrollo neurológico, se ha confirmado en estudios con animales experimentales. Paralelamente a los hallazgos epidemiológicos, los efectos sobre la cognición, la actividad motora y el comportamiento social se demostraron en repetidas ocasiones en los roedores dosificados en edad temprana con concentraciones de OFs que provocan poca o ninguna inhibición de la acetilcolinesterasa (AChE) en el cerebro [10,12]. El momento de la exposición jugó un papel crítico en los objetivos bioquímicos y anatómicos afectados, así como en las específicas alteraciones conductuales y de desarrollo evocadas [12].

Desde la publicación de las revisiones epidemiológicas, se observó una mayor probabilidad de diagnóstico de TEA para los niños nacidos de mujeres que vivían dentro (versus las que vivían más lejos) de 1,5 km de las aplicaciones de plaguicidas OFs en los campos agrícolas; las asociaciones más fuertes fueron para clorpirifos [13]. Otro estudio reciente mostró que concentraciones más altas de metabolitos de OF en la orina materna durante el embarazo se asociaron con rasgos de TEA identificados en la adolescencia [14]. Otros equipos de investigación reportaron que la proximidad de residencias al uso agrícola OF durante el desarrollo fetal se asocia con una reducción en el coeficiente intelectual de los niños a la edad de 7 años [15] y mayores concentraciones de clorpirifos en la sangre del cordón umbilical con temblores de brazo leves a moderados en niños de aproximadamente 11 años de edad [16]. Los riesgos para el neurodesarrollo deteriorado fueron mayores entre los hijos de los trabajadores agrícolas, quienes experimentan exposiciones más altas [17], y los niños con factores genéticos de susceptibilidad que reducen la capacidad para desintoxicar plaguicidas OF [7]. En el mismo estudio que examinó el TEA, moderado a severo retraso en el desarrollo se asoció con aplicaciones cercanas de carbamatos, similares a los plaguicidas OF, pero no con plaguicidas OF [13]. Otros dos estudios, ambos realizados en cohortes urbanas de mayor estatus social y económico, no encontraron asociaciones de metabolitos de plaguicidas OF con puntajes en las pruebas de inteligencia [18, 19]. Aun así, el peso de la evidencia indica claramente que las exposiciones OF durante el desarrollo prenatal son probablemente perjudiciales para la función cerebral.

Una medición precisa de la exposición es crítica en estudios de salud ambiental. Los estudios de plaguicidas OF determinaron exposiciones de varias maneras, que van desde la cuantificación de los metabolitos OF en la orina materna recogida durante el embarazo y la medición directa de clorpirifos en la sangre del cordón umbilical, hasta cuantificar el uso de plaguicidas cercanos geográficamente mediante la vinculación con las direcciones residenciales de la base de datos de California de aplicaciones comerciales de plaguicidas [20,21]. La base de datos de informes de uso de plaguicidas de California, la cual contiene la cantidad específica de plaguicidas y la fecha y la ubicación de cada aplicación, ha sido validada por dos estudios de evaluación de la exposición, los cuales mostraron que la cantidad aplicada en unos pocos días a una semana se correlaciona altamente con concentraciones medidas de aire ambiente en lugares cercanos [22,23]. En la gran mayoría de los estudios revisados, se generaron medidas objetivas (marcadores biológicos y datos de aplicación validados) de acuerdo con protocolos científicamente establecidos y fueron obtenidos independientemente de la evaluación del niño.

**Preocupaciones en exposiciones OF altas y bajas**

Para entender las influencias en el neurodesarrollo infantil, es fundamental distinguir entre los efectos agudos después de exposiciones de alto nivel versus secuelas de exposiciones crónicas inferiores. Como se ha indicado anteriormente, por inhibición de la enzima acetilcolinesterasa, OFs de alto nivel que causan efectos agudos, en algunos casos fatales, en humanos [2]. De hecho, a nivel internacional, las intoxicaciones por plaguicidas causan un gran número de muertes estimadas en 200.000 muertes por año [24], ocurriendo en aproximadamente un 99% en países en desarrollo [25]. Aproximadamente 110.000 muertes por auto-envenenamiento por plaguicidas ocurren cada año en todo el mundo, lo que representa un promedio, entre los países incluidos en el reporte, de 13,7% de todos los suicidios [26], con un amplio rango de 0,9 % en los países europeos de bajos y medianos ingresos a 48,3% para los países de bajos y medianos ingresos de la región del Pacífico occidental.

Grandes cantidades de plaguicidas OF altamente peligrosos son importadas en los países en desarrollo. Por ejemplo, los plaguicidas OF se ubicaron en cuarto lugar entre los 24 grupos químicos de plaguicidas importados en los países centroamericanos [27], por lo que los dos plaguicidas OF importados en la mayor cantidad (terbufos y metamidofos) han sido eliminados por el Convenio de Rotterdam, un acuerdo comercial internacional sobre productos químicos peligrosos, destinado a proteger la salud humana y el medio ambiente [4,28]. Los plaguicidas tóxicos afectan a los trabajadores agrícolas que a menudo reciben poca o ninguna instrucción sobre el uso de sustancias peligrosas, no están provistos de equipo de protección personal, y/o el equipo de aplicación de plaguicidas que se usa no se mantiene correctamente. Además, el uso excesivo, mal uso y los accidentes han provocado la muerte de niños en edad escolar, por ejemplo, en India en 2013, China en 2014 y Bangladesh en 2015, a partir del consumo de comidas con altos niveles de plaguicidas OF[4,24,29,30] .

Tan trágicos como estos envenenamientos agudos, una exposición a plaguicidas OF en la ausencia de envenenamiento abierto no implica que daño neurológico no haya ocurrido –para ambos, niños y adultos [31]. La EPA de los EE. UU. concluyó en 2016 que la literatura epidemiológica existente proporcionó "evidencia suficiente de que hay efectos del neurodesarrollo que se producen a niveles de exposición al clorpirifos por debajo de los requeridos para causar la inhibición de acetilcolinesterasa" [11]. Las exposiciones crónicas de bajo nivel son a menudo ignoradas o rechazadas como como benignas, porque ni la mujer embarazada ni el feto muestran signos o síntomas clínicamente visibles. Además, los déficits de desarrollo no se manifiestan hasta meses o años después. De hecho, el consenso científico es que la inhibición de acetilcolinesterasa (AChE) es poco informativa con respecto a los efectos del desarrollo neurológico en los niños y que los efectos tóxicos de la exposición crónica de bajo nivel ocurren en concentraciones demasiado bajas para inhibir la colinesterasa [1,9]. La evidencia indica que los plaguicidas OF pueden interferir con el desarrollo del cerebro a niveles previamente considerados seguros o intrascendentes.

Por lo tanto, la inhibición de la AChE no se puede utilizar como un biomarcador para identificar las exposiciones a plaguicidas OF como nefastas para el desarrollo. Depender de la inhibición de la AChE para fines normativos oscurece la grave amenaza que representan los plaguicidas OF para el desarrollo temprano del cerebro y representa un enfoque no científico e inadecuado para la evaluación del riesgo para la salud. De hecho, otros efectos parecen probablemente mediar la toxicidad de OF a los sistemas neuronales que son fundamentales para el comportamiento en la infancia y déficits cognitivos. La evidencia toxicológica implica a los plaguicidas OFs en la neuroinflamación, en la señalización del receptor de proteína-quinasa C, en la resistencia a la insulina, en la neurotransmisión dopaminérgica y en la glutamatérgica e interferencia con la síntesis de ADN y el funcionamiento del factor de transcripción nuclear, mecanismos altamente relevantes para el desarrollo del cerebro [12, 32-34].

De hecho, daños aún no descubiertos pueden surgir de futuros seguimientos a personas expuestas en su vida temprana. Los resultados de la exposición del feto parecen ser persistentes con las asociaciones observadas en la infancia media y tardía. Una cohorte mostró repetidamente déficits en memoría, cociente intelectual (CI) y déficits de atención (TDAH) a las edades de 2, 3, 5 y 7 años, mientras que otros estudios mostraron déficits expuestos en el desarrollo mental y el razonamiento en la infancia y en entre las edades de 6 a 9 años (revisado en [8]). Los niños con altas vs bajas concentraciones de clorpirifos en su sangre del cordón umbilical tenían diferencias en el volumen cerebral en las regiones responsables de la atención, el procesamiento del lenguaje receptivo, la cognición social y la regulación de la inhibición [35]. Estas alteraciones neuroanatomías, que potencialmente constituyen una vía desde la exposición a plaguicidas hasta los déficits asociados al comportamiento y en las áreas cognitivas, pueden ser permanentes.

**Regulación de plaguicidas**

Las regulaciones de plaguicidas varían ampliamente en todo el mundo. Al igual que con el uso de plaguicidas, ninguna base de datos ha consolidado esta información para todos los países. La Tabla 1 muestra los datos disponibles sobre 47 insecticidas OF [36] prohibidos por uno o más países, así como el nivel de riesgo para la salud y el número de países que han prohibido cada plaguicidas OF. La mayor base de datos disponible en la regulación gubernamental actual de plaguicidas proporciona datos que cubre 39 de estos 47 insecticidas OF, obtenidos de 106 países fuera de los EE. UU. [37]. En esta base de datos están incluidas prohibiciones totales, junto con denegaciones de aprobación, pero no restricciones. De los 106 países, el 81 % ha regulado uno o más de los 39 insecticidas OF [37]. Los 28 países de la Unión Europea han tomado medidas sobre la mayoría de los plaguicidas OF (33). Entre los países adicionales que han prohibido más de 10 están: los Estados Unidos (26), Camboya (15), China (15), Arabia Saudita (15), Guinea (12), Corea (12) Mauritania (12) y Tailandia (12). Notablemente, imponer regulaciones no significa necesariamente que se cumplan. Además, algunos de los plaguicidas OF más tóxicos que están prohibidos en docenas de países se exportan a otros lugares, a menudo a países en desarrollo y en ocasiones en grandes cantidades, por ejemplo, a Costa Rica y Guatemala [27]. En México, se utilizan al menos una docena de plaguicidas OF que están clasificados como altamente peligrosos por la Organización de las Naciones Unidas para la Alimentación y la Agricultura [38] .

| Tabla 1:  Insecticidas OF, niveles de peligro y número de países que los prohíben | | | | | | |
| --- | --- | --- | --- | --- | --- | --- |
|  | Compuesto ^3^ | Nivel de Peligro:  E = Extremadamente peligroso  H = Altamente peligroso  M = Moderadamente peligroso  S = Ligeramente peligroso  '-' No clasificado H por PAN '**' No clasificado | | | Número de países (fuera de los EE.UU.) que lo han prohibido ^2^ | Organofosforados prohibidos en los EE.UU. designados por X. Todos los otros organofosforados en la lista están actualmente registrados para su uso en los EE.UU. ^3^ |
|  |  | EPA de los EE. UU.^4^ | FAO-OMS ^5^ | PAN ^6^ |  |  |
| 1 | Acefato | M | M | H | 31 |  |
| 2 | Azinfose-metilo | H | H | H | 39 | X |
| 3 | Cadusafos | ** | H | H | 31 |  |
| 4 | Chlorethoxyphos | ** | E | H | 29 |  |
| 5 | Chlorfenvinphos | H | H | H | 35 | X |
| 6 | Clorpyrifos | M | M | H | 2 |  |
| 7 | Clorpyrifos-metilo | ** | S | H | 1 |  |
| 8 | Chlorthiophos ^6,7^ | H | ** | ** | ** | X |
| 9 | Coumaphos | H | H | H | 30 |  |
| 10 | Dichlorfos (diclorvos) | M | H | H | 32 |  |
| 11 | Dialifor / dialifos ^6,7^ | H | ** | ** | ** | X |
| 12 | Diazinon | M | M | H | 30 |  |
| 13 | Dicrotophos | H | H | H | 34 |  |
| 14 | Dimethoate | ** | M | H | 4 |  |
| 15 | Dioxathion ^6,7^ | H | ** | ** | ** | X |
| 16 | Disulfotón | H | E | H | 38 | X |
| 17 | Ethion | M | M | — | 30 | X |
| 18 | Ethoprop  (ethoprophos) | M | E | H | 8 |  |
| 19 | Parathion etílico ^7^ | H | ** | ** | ** | X |
| 20 | Fenamiphos | H | H | H | 6 | X |
| 21 | Fenitrothion | M | M | H | 28 |  |
| 22 | Fenthion | M | M | H | 30 | X |
| 23 | Fonofos (fenophos) ^6^ | H | ** | — | 33 | X |
| 24 | Isazophos ^6,7^ | ** | ** | ** | ** | X |
| 25 | Isofenphos ^6^ | H | ** | — | 29 | X |
| 26 | Malathion | M | S | H | 2 |  |
| 27 | Methamidophos | H | H | H | 49 | X |
| 28 | Metidathion | H | H | H | 34 | X |
| 29 | Parathion metílico | H | E | H | 59 | X |
| 30 | Mevinphos | H | E | H | 37 | X |
| 31 | Monocrotofos | H | H | H | 60 | X |
| 32 | Naled | M | M | H | 28 |  |
| 33 | Oxydemeton-metilo | M | H | H | 30 | X |
| 34 | Phorate | H | E | H | 37 |  |
| 35 | Fosalona | M | M | — | 29 | X |
| 36 | Phosmet ^7^ | M | M | ** | ** |  |
| 37 | Phosphamidon | H | E | H | 49 | X |
| 38 | Phostebupirim ^7^ | ** | ** | ** | ** |  |
| 39 | Pirimifos-metilo ^7^ | M | M | ** | ** |  |
| 40 | Profenofos | M | M | H | 29 | X |
| 41 | Propetamphos | M | H | H | 28 | X |
| 42 | Sulfotepp | H | E | H | 32 | X |
| 43 | Sulprofos ^6,7^ | M | ** | ** | ** | X |
| 44 | Temephos | M | S | H | 28 | X |
| 45 | Terbufos | H | E | H | 34 |  |
| 46 | Tetraclorvinfos | M | ** | H | 28 |  |
| 47 | Triclorfón | M | M | H | 32 |  |
| ^1^ Esta lista de insecticidas organofosforados ha sido tomada de la Oficina de Programas de Plaguicidas de la Agencia de Protección Ambiental de los Estados Unidos (U.S. EPA), “Organophosphorus Cumulative Risk Assessment, 2006 Update " [36] (Tabla ES-1, página 16 OP Pesticides Considered in the 2006 Update of the Cumulative Risk Assessment), de la cual hemos excluido aquellos plaguicidas que no son insecticidas.    ^2^ De la lista consolidada internacional de plaguicidas prohibidos de PAN [37]  ( [http://pan-international.org/pan-international-consolidated-list-of-banned-pesticides/](https://translate.google.com/translate?hl=en&prev=_t&sl=en&tl=es&u=http://pan-international.org/pan-international-consolidated-list-of-banned-pesticides/) ). Los métodos y las fuentes para la recopilación de estos datos se describen en las Notas Explicativas: ( [http://pan-international.org/wp-content/uploads/Consolidated-List-of-Bans-Explanatory-2017April.pdf](https://translate.google.com/translate?hl=en&prev=_t&sl=en&tl=es&u=http://pan-international.org/wp-content/uploads/Consolidated-List-of-Bans-Explanatory-2017April.pdf) ). Esta lista no incluye restricciones, solo prohibiciones o decisiones para no aprobar.    ^3^ Un plaguicida "prohibido" en los EE.UU. se define como un plaguicida para el cual todos los usos registrados han sido prohibidos por la acción final de la EPA e incluye los plaguicidas que se han retirado mediante acuerdos voluntarios entre la industria y la U.S. EPA.  El estado de los organofosfatos que están prohibidos o registrados para su uso en los EE.UU. comunicados comunicación personalde Yu-Ting Guilaran( Director de la División de Reevaluación de Plaguicidas, Oficina de Programas de Plaguicidas, Agencia de Protección Ambiental de EE.UU.) a JBS 12, 13, y 23 de julio, 2018.    ^4^ Clasificación de riesgo [3].    ^5^ Clasificación de riesgo [4]: El concepto y los criterios para los "Plaguicidas Altamente Peligrosos" se describieron inicialmente en el segundo informe de JMPM en 2008, " Informe de la 2da Reunión conjunta FAO / OMS sobre manejo de plaguicidas " (último acceso en julio de 2018) ([http://www.fao.org/fileadmin/templates/agphome/documents/Pests_Pesticides/Code/Report.pdf).](http://www.fao.org/fileadmin/templates/agphome/documents/Pests_Pesticides/Code/Report.pdf).%20) A medida que avanzó la comprensión científica de los mecanismos para la toxicidad de los plaguicidas, estos se han incluido, tal como se describe en la publicación de 2016 del “Código Internacional de Conducta sobre Directrices para el Manejo de Plaguicidas en Plaguicidas Altamente Peligrosos". (<http://apps.who.int/iris/bitstream/handle/10665/205561/9789241510417_eng.pdf;jsessionid=D3B3CCA5B28692A5F3D437B2CF7F0AA0?sequence=1>).  El FAO-OMS JMPM definió los plaguicidas prohibidos así que: "Plaguicidas prohibidos significa un plaguicida cuyos todos sus usos han sido prohibidos por medidas reglamentarias firmes, con el fin de proteger la salud humana o el medio ambiente. Incluye un plaguicida al que se le ha denegado la aprobación para su uso por primera vez, o ha sido retirado de la industria del mercado nacional o de una mayor consideración en el proceso de aprobación nacional, y cuando hay pruebas claras de que dicha medida se tomó con el fin para proteger la salud humana o el medio ambiente".    ^6^ Se lo considera obsoleto o ya no se usa como plaguicidas, de acuerdo con la clasificación recomendada por la OMS de Riesgos de Plaguicidas, 2010.  ^7^ No incluido en la base de datos del PAN.  Abreviaciones: EPA, Agencia de Protección Ambiental de Estados Unidos; FAO-OMS: Organización de las Naciones Unidas para la Alimentación Agricultura y la Agricultura y la Organizcion Mundial de la Salud; JMPM: Reunión conjunta FAO-OMS sobre manejo de plaguicidas; PAN, Red de Acción de Plaguicidas | | | | | | |

Dentro de los EE.UU., la EPA regula plaguicidas bajo dos estatutos superpuestos—la ley Federal de Alimentos, Medicamentos y Cosméticos (FFDCA) y la Ley Federal de Insecticidas, Fungicidas y Rodenticidas (FIFRA). Muchos de los insecticidas prohibidos en los EE. UU. fueron autorizados inicialmente antes de 1970 cuando la evaluación de salud y seguridad requerida era mínima y antes de que se formara la EPA de EE. UU. Como resultado de la legislación en la década de 1970 que requieren mayores estudios de salud y seguridad, se alcanzaron acuerdos voluntarios entre fabricantes y la EPA para eliminar o reducir progresivamente registros de algunos plaguicidas, incluidos 18 insecticidas OF.

En 1996, la Ley de Protección de la Calidad de los Alimentos (FQPA) modificó la FIFRA y FFDCA al exigir que la EPA incluyera factores de seguridad adicionales para proteger a los niños por su mayor exposición y susceptibilidad [39]. Los niños tienen mayor carga corporal de plaguicidas debido al mayor consumo de alimentos, agua y aire que los adultos por unidad de su peso corporal; exploran el mundo a través de comportamientos introduciendo cosas a la boca; y frecuentemente gatean o juegan en pisos donde plaguicidas y otros químicos tóxicos permanecen. Aún más, la susceptibilidad durante los primeros años surge en parte de unos sistemas inmaduros de enzimas de detoxificación, incluido paraoxonasa 1 (PON1) [7, 40, 41]. Según la FQPA, la EPA debe mostrar que existe una certeza razonable de que la exposición total a los plaguicidas no causará daños, incluyendo todas las exposiciones dietéticas anticipadas y todas las demás exposiciones para las cuales existe información confiable.

Después del aprobación  FQPA, el uso de plaguicidas OF en todos los sectores del mercado disminuyeron, más del 70% bajó de 70 millones de libras por año (lbs /año) en el 2000 a alrededor de 20 millones de lbs/año en el 2012 (según los datos disponibles más recientes) [6]. Para el año 2002, el uso no agrícola de plaguicidas fue eliminado progresivamente por un acuerdo entre la EPA y los fabricantes de plaguicidas, en base al resultado de la evaluación de riesgos de la EPA de clorpirifos y diazinón que muestran inaceptablemente altos riesgos para los residentes, especialmente los niños, del uso residencial de plaguicidas [42,43]. El volumen de plaguicidas organofosforados utilizados en los alimentos communmente consumido por los niños, como las frutas, disminuyó en un 57% entre 1994 y 2004, de 28 a 12 millones de libras (12.701 a 5.443 toneladas métricas) de ingrediente activo aplicado anualmente [44]. Esta acción resultó en reducciones dramáticas en concentraciones de OF en la sangre y orina entre la población de EE. UU. [45]. Sin embargo, el uso agrícola de plaguicidas OF continua contribuyendo a la exposición de los trabajadores agrícolas, sus familias[15], los residentes en hogares,  niños en las escuelas y otras personas presentes cerca de las tierras de cultivo[23], así como la contaminación de alimentos y del agua potable que afecta una amplia población.

En 2016, la EPA de los EE. UU. concluyó que la exposición a clorpirifos, el insecticida OF de uso más común en los EE. UU., ya sea ​​por alimentos o solo por agua potable podrían causar exposiciones inaceptablemente altas a la población, y determinó que algunas mujeres en edad reproductiva, los bebés, y los niños consumieron niveles de clorpirifos sustancialmente por encima del nivel aceptable para estas etapas vulnerables de la vida [11]. La EPA también identificó numerosos escenarios que podrían resultar en exposiciones peligrosas para los trabajadores agrícolas y espectadores. Por estas razones, según lo exige la ley, la EPA propuso revocar todos los estándares (llamados tolerancias) que permiten residuos de clorpirifos en los alimentos. La revocación de estas tolerancias esencialmente prohibiría este OF en cultivos alimenticios [11].  Sin embargo, en marzo de 2017, a pesar de la evidencia abrumadora del daño, y contrariamente a las propias evaluaciones de riesgo de la EPA, la EPA de la administración de Trump anunció que "la ciencia que aborda los efectos del neurodesarrollo sigue sin resolverse, y que posterior evaluación de la ciencia ... [por lo tanto] está garantizado para lograr una mayor certeza en cuanto a si existe la posibilidad de que se produzcan efectos adversos en el desarrollo neurológico de las exposiciones humanas actuales al clorpirifos", concluyendo que la EPA no cancelaría ningún uso de clorpirifos[46]. Esta acción retrasaría la acción regulatoria potencial hasta octubre de 2022. Sin embargo, el 9 de agosto de 2018, la Corte de Apelaciones de los Estados Unidos del Noveno Circuito ordenó a la EPA finalizar la prohibición de clorpirifos en un plazo de 60 días, incluyendo la prohibición de todas las ventas en EE. UU. y la prohibición de alimentos contaminados por el insecticida que lluege al mercado en los EE.UU.. La corte basó su decisión en los hallazgos de la EPA de 2016 en que el plaguicida no cumple con las normas de seguridad federales y era particularmente dañino para bebés y niños. En septiembre de 2018, la EPA presentó una petición para una nueva audiencia del caso de clorpirifos.

**Recomendaciones**

En 2014, la Academia Americana de Pediatría pidió que los pediatras y los gobiernos reconozcan y reduzcan las exposiciones a plaguicidas a través de la educación, el etiquetado de plaguicidas, la vigilancia de la salud pública y la acción reguladora [47]. En 2016, un grupo independiente de científicos y profesionales de la salud publicaron el Proyecto TENDR Declaración de Consenso como un llamado nacional a la acción para reducir significativamente la exposición a los productos químicos, incluidos los plaguicidas OF, que se ha identificado que ponen a los niños en los Estados Unidos, y probablemente en todo el mundo, en mayor riesgo de trastornos del neurodesarrollo [48]. El Proyecto TENDR concluyó que la evidencia de riesgos significativos para el neurodesarrollo de los niños a causa de la exposición a plaguicidas OF justifica una fuerte acción reguladora. En 2017, un informe de las Naciones Unidas sobre el Derecho a Alimentos pidió cambios en las prácticas agrícolas para garantizar una alimentación segura, libre de plaguicidas y cualitativamente adecuada [24]. Para lograr el objetivo de reducir la exposición a los insecticidas OF, por lo tanto, proponemos un plan de acción para el gobierno, instituciones u organizaciones médicas y de salud pública, y entidades agrícolas. Nuestras recomendaciones se detallan en el cuadro 1 contiguo. Estos pasos notablemente reducirían la exposición a plaguicidas organofosforados durante la etapa prenatal y en la infancia.

Cuadro 1: Recomendaciones para avanzar hacia la eliminación de las exposiciones humanas a los plaguicidas OF.

Recomendamos las siguientes acciones por parte de los gobiernos:

• Que los gobiernos nacionales y estatales o provinciales, a nivel mundial eliminen gradualmente el uso de todos los organofosforados en la agricultura;

• Que los gobiernos nacionales, estatales o provinciales, a nivel mundial prohíban el uso no agrícola de todos los organofosforados, incluidos los productos para el hogar;

• Que la EPA de los EE.UU. revoque todas las tolerancias de los alimentos para el clorpirifos, como la agencia propuso anteriormente;

• Que la EPA de los EE.UU. y gobiernos estatales eliminen gradualmente el uso de todos los demás organofosforados en agricultura;

• Que la EPA de los EE.UU. prohíba los usos de control de plaguicidas no agrícolas de los pocos organofosforados restantes;

• Mientras tanto, que las agencias nacionales, estatales y locales tomen medidas para reducir la exposición humana (por ejemplo, requieran notificación previa a los residentes y escuelas cercanas antes de las aplicaciones de plaguicidas organofosforados; implementan restricciones en los métodos de aplicación, tales como fumigación aéreal y ráfagas de aire para reducir la deriva exposiciones y para proteger el agua y sitios sensibles como hogares y escuelas);

• Que las agencias nacionales, estatales y locales realicen un monitoreo regular de las cuencas hidrográficas para garantizar que los organofosforados no continúen contaminando lagos, ríos y arroyos, incluidos los que son fuentes de agua potable, y que implementen un monitoreo específico del agua potable;

• Que las agencias nacionales y estatales establezcan un programa integral efectivo de uso de plaguicidas y reporte de enfermedades, ya sea a nivel nacional, o a través de programas estatales coordinados.

Recomendamos que las escuelas de medicina, los programas de salud pública y las asociaciones de atención médica:

• Organicen cursos de educación médica continua para educar a los proveedores de atención médica sobre los efectos agudos y crónicos de la exposición a sustancias químicas tóxicas, que incluyen: cómo reconocer y tratar a los niños que recibieron altas exposiciones organofosforados; cómo aconsejar a las mujeres embarazadas y los padres de niños pequeños sobre las medidas que pueden tomar para evitar la exposición a los plaguicidas de los tratamientos de piojos, pulgas y garrapatas [49], productos de jardinería, y aplicaciones en las tierras agrícolas cercanas, campo de golf, escuelas y centros comerciales; cómo limpiar apropiadamente los residuos potenciales de plaguicidas de las frutas y verduras y identificar cuales productos contienen los niveles más altos;

• Eduquen a los proveedores de salud en el informe necesario de envenenamiento por plaguicidas a la vigilancia estatal;

• Alienten a las escuelas de enfermería y medicina a incorporar planes de estudios sobre los peligros ambientales que incluyen plaguicidas y consejos médicos para incluir la salud ambiental en sus exámenes.

Recomendamos que las entidades agrícolas:

• Brinden mayor capacitación a los trabajadores, en los idiomas más apropiados y en los niveles educativos pertinentes, en el manejo y aplicación de plaguicidas y en las normas de protección de los trabajadores. En los EE.UU., esto significa que los entrenamientos estándares de Protección al Trabajador de EPA con el requerido frecuencia;

• Eduquen los trabajadores sobre cómo evitar la exposición a sus familias en el hogar;

• Instituyan enfoques amigables con el medio ambiente para controlar los parásitos - manejo integrado de parásitos (MIP) - con el objetivo de eliminar o minimizar los químicos tóxicos en nuestras fuentes de alimentos.

Se han tomado medidas ejemplares en varios niveles gubernamentales. A nivel multinacional, la UE optó por *no aprobar* cerca de 200 plaguicidas de los cuales más de 20 son OF, y varios países individuales han instituido prohibición de OF tales como diclorvos, metamidofos y metil paratión [37]. En los EE.UU., California ha tomado medidas para limitar el uso agrícola de plaguicidas cerca de escuela e instalaciones de cuidado de niños cuando los niños están presentes [50] y Hawái, recientemente, prohibió la distribución, venta, transporte y uso de cualquier plaguicida que contenga clorpirifos como un ingrediente activo [51].

En la reducción del uso de plaguicidas OF, los efectos tóxicos de sustituto o los productos químicos de reemplazo requieren escrutinio. Plaguicidas piretroides han reemplazado OF como la principal clase de insecticidas en los productos de control de plagas residenciales, pero estudios recientes de laboratorio de roedores y estudios epidemiológicos sugieren que la exposición a los plaguicidas piretroides durante la etapa prenatal también puede aumentar el riesgo de resultados adversos del neurodesarrollo, el comportamiento y las emociones negativas [13, 52-54]. Los plaguicidas neonicotinoide son ahora la clase de más rápido crecimiento de los insecticidas utilizados en los cultivos en los EEE. UU. [55]; son persistentes en las plantas, la tierra y el agua, y altamente tóxicos para los invertebrados, incluidas las especies acuáticas en peligro de extinción, las abejas y otros insectos benéficos [56]. Además, está bien documentado que los impactos del uso amplio y sistémico de plaguicidas han tenido consecuencias ecológicas negativas significativas que afectan los hábitats terrestres, acuáticos, humedales, marinos y bentónicos, y que presentan riesgos para el funcionamiento y la resiliencia de los ecosistemas.

¿Cuáles son las alternativas, si los plaguicidas sintéticos que no sean OF también son neurotóxicos?

La agricultura representa la gran mayoría del uso de plaguicidas OF, que incluye tanto para la producción agrícola y como para la ganadera. Se necesita una implementación generalizada de MIP para reducir este uso. MIP es una estrategia de gestión de plagas de riesgo reducido que hace énfasis en la inspección, monitoreo, prevención y control de plagas utilizando métodos menos tóxicos, incluyendo (AGRI) prácticas culturales como inter-cultivo (crecimiento de dos o más cultivos en estrecha proximidad, que puede reducir la susceptibilidad a las enfermedades y plagas), rotación de cultivos y cultivos de cobertura (para reducir la erosión del suelo y mejorar la salud de la tierra), controles físicos como trampas o aspiradoras de insectos, manejo del hábitat que fomenta insectos beneficiosos y control biológico, como la liberación de avispas parasitarias a controlar los áfidos, con plaguicidas utilizados solo como último recurso. Cuando se usan, los plaguicidas menos tóxicos se elijan primero, como los materiales aprobados para la agricultura orgánica (p. ej., *bacillus thuringiensis* para controlar los lepidópteros) [57].

Aunque estrategias de MIP no prohíben, en principio, el uso de OF y otros plaguicidas neurotóxicos, estos materiales de mayor riesgo sirven como último recurso y deben aplicarse de manera que protejan la salud humana y ambiental. La mayoría de los cultivos producidos con plaguicidas organofosforados también son producidos orgánicamente, y comprueban que los plaguicidas organofosforados no son esenciales [58]. Algunas plagas recalcitrantes pueden ser difíciles de manejar con plaguicidas menos tóxicos, que en algunos casos puede resultar en un menor rendimiento o mayores costos de producción, reduciendo competitividad. Investigaciones recientes, sin embargo, indica que los rendimientos de cultivos a partir de los sistemas de producción alternativos y orgánicos están aumentando y en algunos casos iguales a los rendimientos convencionales [59]; estos enfoques adicionalmente probablemente reducirían los costos externos para la salud pública y el medio ambiente [60]. Para asegurar que los agricultores no están en peligro de aumento de los costos y los márgenes de ganancia más delgados, muchos comercios agrícolas y organizaciones políticas recomiendan un mayor apoyo gubernamental para la investigación de extensión y alcance necesarios para apoyar las transiciones a materiales menos tóxicos [61].

La salud pública, es un segundo uso de pesticidas OF, que representa una pequeña fracción de sus aplicaciones. Por ejemplo, los plaguicidas OF se usan para el control de mosquitos y otros vectores, para prevenir enfermedades transmitidas por vectores como el virus del Zika o el virus del Nilo Occidental. No recomendamos cambios abruptos en el manejo de plagas que aumenten el riesgo de exposición a estos virus. Nosotros abogamos por una mayor financiación para una mejor comprensión de la ecología y la biología de estos y otros vectores y las enfermedades que causan y métodos alternativos para el control de ellos sin el uso de OF u otros plaguicidas neurotóxicos. El ejemplo histórico de la mosca mediterránea de la fruta en California, una plaga agrícola invasiva grave, proporciona un modelo para la aplicación de vectores de enfermedades. A principios de la década de 1990, los funcionarios estatales utilizaron helicópteros para pulverizar malatión en áreas residenciales donde residían más de dos millones de personas [62]. Preocupaciones  subsiguientes [63] dieron como resultado el desarrollo de un programa integral de liberación de moscas de la fruta estéril que, combinado con tratamientos puntuales que a menudo usan plaguicidas aprobados orgánicamente, ha controlado con éxito infestaciones sin necesidad de aplicaciones de plaguicidas OF en amplias zonas residenciales [64, 65]. También se deben considerar estrategias similares para las nuevas especies invasoras, como la mosca manchada, que actualmente amenaza los ecosistemas y la agricultura del este de los EE. UU.. Gestión integrada de vectores favorecería el uso de opciones menos tóxicas.

Las aplicaciones de plaguicidas tanto estructurales como en interiores y jardines, la tercera categoría de usos de OF, pueden dar como resultado altas exposiciones*.* Dichlorvos, un OF ya prohibido en muchos países, aun es permitido en estructuras interiores por el gobierno de Estados Unidos para los insectos voladores. De manera similar, el malatión aún se vende para uso paisajista y jardines. Debido a los riesgos de efectos adversos para la salud a causa a la exposición crónica, de bajo nivel, e intoxicación aguda de los consumidores en los EE.UU. que se han reportado [66], se recomienda que todos los restantes OFs usados estructurales, interiores y jardines se eliminen de inmediato, especialmente en entornos donde hay niños presentes. Principios básicos de MIP se deben aplicar en estos entornos, incluyendo la exclusión de plagas (es decir, pantallas) y trampas.

Para preservar la salud y la sostenibilidad, tanto en interiores como en exteriores el manejo de plagas debe depender en alternativas que no sean tóxicas o que sean menos tóxicas; al mismo tiempo, la agricultura necesita un apoyo más fuerte para avanzar hacia un enfoque sistémico que minimice el uso de plaguicidas neurotóxicos a la vez que proporcione alimentos saludables y sostenibilidad económica para los agricultores. El Informe sobre el Derecho a la Alimentación del Relator Especial para la Asamblea General de las Naciones Unidas articula una filosofía similar: para reducir o eliminar con éxito el uso de plaguicidas peligrosos, los esfuerzos de la comunidad internacional deberán abordar los factores ecológicos, sociales y económicos actualmente integrados en las políticas agrícolas. A nivel nacional, esto requerirá una agricultura desafiante dependiente de agroquímicos para reestructurarse y buscar las alternativas más seguras posibles [24]. Nos unimos a la Academia Americana de Pediatría y la ONU en recomendar estrecha vigilancia de intoxicaciones por plaguicidas, incentivos para alternativas no químicas para el control de plagas, la vigilancia de las fuentes de agua y alimentos de los plaguicidas, y la aplicación del derecho del público a conocer a través de la divulgación completa, etiquetado y otras comunicaciones para formulaciones de plaguicidas y para residuos en alimentos, agua y en otros lugares. Por último, creemos que es una responsabilidad ética y social de la sociedad civil para la profesión médica, y para la industria agrícola para diseminar ampliamente al público en general lo que se conoce acerca de las fuentes de exposición a los plaguicidas y sus impactos adversos sobre la salud, y para desarrollar programas de formación en agroecología con el fin de lograr un cambio de paradigma en la producción de alimentos.

Expresiones de gratitud:

Estamos agradecidos por el apoyo de Maureen Swanson. 

REFERENCIAS:

1. Costa LG. Organophosphorus Compounds at 80: Some Old and New Issues. Toxicol Sci. 2018;162(1):24-35. Epub 2017/12/12. doi: 10.1093/toxsci/kfx266. PubMed PMID: 29228398.

2. Soltaninejad K, Shadnia S. History of the Use and Epidemiology of Organophosphorus Poisoning. In: Basic and Clinical Toxicology of Organophosphorus Compounds Editors: Mahdi Balali-Mood, Mohammad Abdollahi, Springer. London, 2014.

3. Roberts J, Reigart J. Chapter 5. Organophosphates. Recognition and Management of Pesticide Poisonings. Sixth ed. Washington, D.C.: U.S. ENvironmental Protection Agency, Office of Pesticide Programs; 2013. Available at <http://www2.epa.gov/pesticide-worker-safety>

4. World Health Organization International Programme on Chemical Safety. The WHO Recommended Classification of Pesticides by Hazard and Guidelines to Classification 2009. Geneva, Switzerland: 2009. Cited Sept 1, 2018. (<http://www.who.int/ipcs/publications/pesticides_hazard_2009.pdf?ua=1>)

5. Pesticides Use Data [Internet]. 2017 [cited June 28, 2018]. Available from: <http://www.fao.org/faostat/en/#data/RP>.

6. U.S. EPA. Report of Pesticide Industry Sales and Usage, 2008-2012 Market Estimates. United States Environmental Protection Agency, Washington, DC; 2017 [cited July 11, 2018]. Available from: https://www.epa.gov/sites/production/files/2017-01/documents/pesticides-industry-sales-usage-2016_0.pdf

7. Gonzalez-Alzaga B, Lacasana M, Aguilar-Garduno C, Rodriguez-Barranco M, Ballester F, Rebagliato M, et al. A systematic review of neurodevelopmental effects of prenatal and postnatal organophosphate pesticide exposure. Toxicol Lett. 2014;230(2):104-21. doi: 10.1016/j.toxlet.2013.11.019. PubMed PMID: 24291036.

8. Koureas M, Tsakalof A, Tsatsakis A, Hadjichristodoulou C. Systematic review of biomonitoring studies to determine the association between exposure to organophosphorus and pyrethroid insecticides and human health outcomes. Toxicol Lett. 2012;210(2):155-68. doi: 10.1016/j.toxlet.2011.10.007. PubMed PMID: 22020228.

9. Munoz-Quezada MT, Lucero BA, Barr DB, Steenland K, Levy K, Ryan PB, et al. Neurodevelopmental effects in children associated with exposure to organophosphate pesticides: a systematic review. Neurotoxicology. 2013;39:158-68. doi: 10.1016/j.neuro.2013.09.003. PubMed PMID: 24121005; PubMed Central PMCID: PMC3899350.

10. U.S. EPA. EPA Revised Human Health Risk Assessment on Chlorpyrifos. December 2014. Docket ID EPA-HQ-OPP-2008-0850. Available from: <http://www.epa.gov/ingredients-used-pesticide-products/revised-human-health-risk-assessment-chlorpyrifos>

11. U.S. EPA. Chlorpyrifos: Revised Human Health Risk Assessment for Registration Review. US Environmental Protection Agency Washington, DC; 2016. Document ID: EPA-HQ-2015-0653-0454. Available from: <https://www.regulations.gov/document?D=EPA-HQ-OPP-2015-0653-0454>.

12. Abreu-Villaca Y, Levin ED. Developmental neurotoxicity of succeeding generations of insecticides. Environ Int. 2017;99:55-77. Epub 2016/12/03. doi: 10.1016/j.envint.2016.11.019. PubMed PMID: 27908457; PubMed Central PMCID: PMC5285268.

13. Shelton JF, Geraghty EM, Tancredi DJ, Delwiche LD, Schmidt RJ, Ritz B, et al. Neurodevelopmental disorders and prenatal residential proximity to agricultural pesticides: the CHARGE study. Environ Health Perspect. 2014;122(10):1103-9. doi: 10.1289/ehp.1307044. PubMed PMID: 24954055; PubMed Central PMCID: PMC4181917.

14. Sagiv SK, Harris MH, Gunier RB, Kogut KR, Harley KG, Deardorff J, et al. Prenatal Organophosphate Pesticide Exposure and Traits Related to Autism Spectrum Disorders in a Population Living in Proximity to Agriculture. Environ Health Perspect. 2018;126(4):047012. Epub 2018/04/28. doi: 10.1289/EHP2580. PubMed PMID: 29701446.

15. Gunier RB, Bradman A, Harley KG, Kogut K, Eskenazi B. Prenatal Residential Proximity to Agricultural Pesticide Use and IQ in 7-Year-Old Children. Environ Health Perspect. 2017;125(5):057002. doi: 10.1289/EHP504. PubMed PMID: 28557711.

16. Rauh VA, Garcia WE, Whyatt RM, Horton MK, Barr DB, Louis ED. Prenatal exposure to the organophosphate pesticide chlorpyrifos and childhood tremor. Neurotoxicology. 2015;51:80-6. doi: 10.1016/j.neuro.2015.09.004. PubMed PMID: 26385760.

17. Engel SM, Bradman A, Wolff MS, Rauh VA, Harley KG, Yang JH, et al. Prenatal Organophosphorus Pesticide Exposure and Child Neurodevelopment at 24 Months: An Analysis of Four Birth Cohorts. Environ Health Perspect. 2016;124(6):822-30. doi: 10.1289/ehp.1409474. PubMed PMID: 26418669; PubMed Central PMCID: PMC4892910.

18. Cartier C, Warembourg C, Le Maner-Idrissi G, Lacroix A, Rouget F, Monfort C, et al. Organophosphate Insecticide Metabolites in Prenatal and Childhood Urine Samples and Intelligence Scores at 6 Years of Age: Results from the Mother-Child PELAGIE Cohort (France). Environ Health Persp. 2016;124(5):674-80. doi: 10.1289/ehp.1409472. PubMed PMID: WOS:000377077000025.

19. Donauer S, Altaye M, Xu Y, Sucharew H, Succop P, Calafat AM, et al. An Observational Study to Evaluate Associations Between Low-Level Gestational Exposure to Organophosphate Pesticides and Cognition During Early Childhood. Am J Epidemiol. 2016;184(5):410-8. Epub 2016/08/20. doi: 10.1093/aje/kwv447. PubMed PMID: 27539379; PubMed Central PMCID: PMC5013882.

20. California Department of Pesticide Regulation. Pesticide Use Reporting (PUR) (Home Page) [cited August 2017]. Available from: <https://www.cdpr.ca.gov/docs/pur/purmain.htm>

21. California Department of Pesticide Regulation. Overview of Pesticide Use Reporting (PUR). https://www.cdpr.ca.gov/docs/pur/purovrvw/tabofcon.htm

22. Harnly M, McLaughlin R, Bradman A, Anderson M, Gunier R. Correlating agricultural use of organophosphates with outdoor air concentrations: a particular concern for children. Environ Health Perspect. 2005;113(9):1184-9. Epub 2005/09/06. PubMed PMID: 16140625; PubMed Central PMCID: PMC1280399.

23. Wofford P, Segawa R, Schreider J, Federighi V, Neal R, Brattesani M. Community air monitoring for pesticides. Part 3: using health-based screening levels to evaluate results collected for a year. Environ Monit Assess. 2014;186(3):1355-70. doi: 10.1007/s10661-013-3394-x. PubMed PMID: 24370859.

24. United Nations. Report of the U.N. Special Rapporteur on the Right to Food. Human Rights Council 34th Session; 2017.

25. Goldman LR. Childhood Pesticide Poisoning: Information for Advocacy and Action. Geneva, Switzerland: United Nations Environment Programme and WHO, 2004.

26. Mew EJ, Padmanathan P, Konradsen F, Eddleston M, Chang SS, Phillips MR, et al. The global burden of fatal self-poisoning with pesticides 2006-15: Systematic review. J Affect Disord. 2017;219:93-104. Epub 2017/05/24. doi: 10.1016/j.jad.2017.05.002. PubMed PMID: 28535450.

27. Bravo V, Rodriguez T, van Wendel de Joode B, Canto N, Calderon GR, Turcios M, et al. Monitoring pesticide use and associated health hazards in Central America. Int J Occup Environ Health. 2011;17(3):258-69. Epub 2011/09/13. doi: 10.1179/107735211799041896. PubMed PMID: 21905395.

28. Rotterdam Convention on the Prior Informed Consent Procedure for Certain Hazardous Chemicals and Pesticides in International Trade, (2004, Revised 2015).

29. Reuters. Pesticide Found in Meals That Killed Indian Children, Official Says. New York Times, July 2013. <https://www.nytimes.com/2013/07/22/world/asia/pesticide-found-in-meals-that-killed-india-children-official-says.html>

30. Roy P, Karmakar K. Pesticide in litchi kills kids, again. The Daily Star, June 2015. <https://www.thedailystar.net/frontpage/pesticide-litchi-kills-kids-again-103651>

31. Starks SE, Hoppin JA, Kamel F, Lynch CF, Jones MP, Alavanja MC, et al. Peripheral nervous system function and organophosphate pesticide use among licensed pesticide applicators in the Agricultural Health Study. Environ Health Perspect. 2012;120(4):515-20. Epub 2012/01/21. doi: 10.1289/ehp.1103944. PubMed PMID: 22262687; PubMed Central PMCID: PMC3339452.

32. Banks CN, Lein PJ. A review of experimental evidence linking neurotoxic organophosphorus compounds and inflammation. Neurotoxicology. 2012;33(3):575-84. doi: 10.1016/j.neuro.2012.02.002. PubMed PMID: 22342984; PubMed Central PMCID: PMC3358519.

33. Bjorling-Poulsen M, Andersen HR, Grandjean P. Potential developmental neurotoxicity of pesticides used in Europe. Environ Health. 2008;7:50. doi: 10.1186/1476-069X-7-50. PubMed PMID: 18945337; PubMed Central PMCID: PMC2577708.

34. Lasram MM, Dhouib IB, Annabi A, El Fazaa S, Gharbi N. A review on the molecular mechanisms involved in insulin resistance induced by organophosphorus pesticides. Toxicology. 2014;322:1-13. doi: 10.1016/j.tox.2014.04.009. PubMed PMID: 24801903.

35. Rauh VA, Perera FP, Horton MK, Whyatt RM, Bansal R, Hao X, et al. Brain anomalies in children exposed prenatally to a common organophosphate pesticide. Proc Natl Acad Sci U S A. 2012;109(20):7871-6. doi: 10.1073/pnas.1203396109. PubMed PMID: 22547821; PubMed Central PMCID: PMC3356641.

36. U.S. EPA Office of Pesticide Programs. Organophosphorus Cumulative Risk Assessment, 2006 Update. 2006. p. 522 (190 without the appendices).

37. Pesticide Action Network. International Consolidated List of Banned Pesticides 2017 [Accessed July 6, 2018]. Available from: http://pan-international.org/pan-international-consolidated-list-of-banned-pesticides/

38. Bejarano Gonzales F, editor. Highly Hazardous Pesticides in Mexico. 1st English Edition. RAPAM; 2018. Texcoco, Mexico. Available from: <http://www.academia.edu/36923833/Highly_Hazardous_Pesticides_in_Mexico01.pdf>

39. Food Quality Protection Act of 1996, Pub. L. No. 104-170, 1996. 104^th^ Congress. 110 STAT. 1489. Available: <http://frwebgate.access.gpo.gov/cgi-bin/getdoc.cgi?dbname=104_cong_public_laws&docid=f:publ170.104>

40. Engel SM, Wetmur J, Chen J, Zhu C, Barr DB, Canfield RL, et al. Prenatal exposure to organophosphates, paraoxonase 1, and cognitive development in childhood. Environ Health Perspect. 2011;119(8):1182-8. doi: 10.1289/ehp.1003183. PubMed PMID: 21507778; PubMed Central PMCID: PMC3237356.

41. Huen K, Harley K, Brooks J, Hubbard A, Bradman A, Eskenazi B, et al. Developmental changes in PON1 enzyme activity in young children and effects of PON1 polymorphisms. Environ Health Perspect. 2009;117(10):1632-8. Epub 2009/12/19. doi: 10.1289/ehp.0900870. PubMed PMID: 20019917; PubMed Central PMCID: PMC2790521.

42. U.S. EPA. Diazinon Revised Risk Assessment and Agreement with Registrants; 2001.

43. U.S. EPA. Chlorpyrifos Revised Risk Assessment and Agreement with Registrants. Environmental Protection Agency, Washington, DC; 2001. Available from: <http://www.ibiblio.org/london/NAFEX/message-archives/old/pdf00000.pdf>

44. Edwards D, U.S. EPA. Reregistration and Tolerance Reassessment. U.S. Environmental Protection Agency; 2006.

45. Clune AL, Ryan PB, Barr DB. Have regulatory efforts to reduce organophosphorus insecticide exposures been effective? Environ Health Perspect. 2012;120(4):521-5. doi: 10.1289/ehp.1104323. PubMed PMID: 22251442; PubMed Central PMCID: PMC3339465.

46. U. S. EPA. Federal Register for Wednesday, April 5, 2017 (FR 16581) (FRL–9960–77) EPA–HQ–OPP–2007–1005; Chlorpyrifos; Order Denying PANNA and NRDC’s Petition To Revoke Tolerances. US Environmental Protection Agency. Washington, DC; 2017. Docket ID EPA-HQ-OPP-2007-1005-0100. Available from: <https://www.regulations.gov/document?D=EPA-HQ-OPP-2007-1005-0100>

47. Roberts JR, Karr CJ, Council On Environmental H. Pesticide exposure in children. Pediatrics. 2012;130(6):e1765-88. Epub 2012/11/28. doi: 10.1542/peds.2012-2758. PubMed PMID: 23184105; PubMed Central PMCID: PMC5813803.

48. Bennett D, Bellinger DC, Birnbaum LS, Bradman A, Chen A, Cory-Slechta DA, et al. Project TENDR: Targeting Environmental Neuro-Developmental Risks The TENDR Consensus Statement. Environ Health Perspect. 2016;124(7):A118-22. doi: 10.1289/EHP358. PubMed PMID: 27479987; PubMed Central PMCID: PMC4937840.

49. Centers for Disease Control and Prevention. Head Lice – General Guidelines. Centers for Disease Control and Prevention. Atlanta, Georgia; 2016. Available from: <http://www.cdc.gov/parasites/lice/head/treatment.html>

50. Regulation to Address Pesticides Used Near Schools and Child Day Care Facilities, California Code of Regulations, Title 3. Sect. Sections 6690-6692 (2018).

51. Reese D. Hawaii Becomes First State to Ban Popular Pesticide; 2018. Available from: <https://www.courthousenews.com/hawaii-becomes-first-state-to-ban-popular-pesticide/>

52. Eskenazi B, An S, Rauch SA, Coker ES, Maphula A, Obida M, et al. Prenatal Exposure to DDT and Pyrethroids for Malaria Control and Child Neurodevelopment: The VHEMBE Cohort, South Africa. Environ Health Perspect. 2018;126(4):047004. Epub 2018/04/13. doi: 10.1289/EHP2129. PubMed PMID: 29648420.

53. Furlong MA, Barr DB, Wolff MS, Engel SM. Prenatal exposure to pyrethroid pesticides and childhood behavior and executive functioning. Neurotoxicology. 2017;62:231-8. Epub 2017/08/16. doi: 10.1016/j.neuro.2017.08.005. PubMed PMID: 28811173; PubMed Central PMCID: PMC5623638.

54. Richardson JR, Taylor MM, Shalat SL, Guillot TS 3rd, Caudle WM, Hossain MM, et al. Developmental pesticide exposure reproduces features of attention deficit hyperactivity disorder. FASEB J. 2015;29(5):1960-72. doi: 10.1096/fj.14-260901. PubMed PMID: 25630971; PubMed Central PMCID: PMC4415012.

55. Jeschke P, Nauen R, Schindler M, Elbert A. Overview of the status and global strategy for neonicotinoids. J Agric Food Chem. 2011;59(7):2897-908. doi: 10.1021/jf101303g. PubMed PMID: 20565065.

56. The Task Force on Systemic Pesticides (TFSP). Worldwide Integrated Assessment of the Impacts of Systemic Pesticides on Biodiversity and Ecosystems; 2015. Available from: <http://www.tfsp.info/worldwide-integrated-assessment/>

57. Organic Materials Review Institute (OMRI). OMRI Products LIst Eugene, OR [6/27/18]. Available from: <https://www.omri.org/omri-lists>

58. National Agricultural Statistics Service. Certified Organic Survey 2016 Summary. Washington, D.C. : U.S. Department of Agriculture; 2017.

59. Ponisio LC, M'Gonigle LK, Mace KC, Palomino J, de Valpine P, Kremen C. Diversification practices reduce organic to conventional yield gap. Proc Biol Sci. 2015;282(1799):20141396. Epub 2015/01/27. PubMed PMID: 25621333; PubMed Central PMCID: PMC4286047.

60. Bellinger DC. A strategy for comparing the contributions of environmental chemicals and other risk factors to neurodevelopment of children. Environ Health Perspect. 2012;120(4):501-7. Epub 2011/12/21. doi: 10.1289/ehp.1104170. PubMed PMID: 22182676; PubMed Central PMCID: PMC3339460.

61. Organic Trade Association and The Organic Center applaud bipartisan bill to invest in organic ag research [Internet]. Organic Trade Association; 2017. Available from: <https://www.ota.com/news/press-releases/19672>

62. Bradman MA, Harnly ME, Goldman LR, Marty MA, Dawson SV, Dibartolomeis MJ. Malathion and malaoxon environmental levels used for exposure assessment and risk characterization of aerial applications to residential areas of southern California, 1989-1990. J Expo Anal Environ Epidemiol. 1994;4(1):49-63. Epub 1994/01/01. PubMed PMID: 7894268.

63. Thomas DC, Petitti DB, Goldhaber M, Swan SH, Rappaport EB, Hertz-Picciotto I. Reproductive outcomes in relation to malathion spraying in the San Francisco Bay Area, 1981-1982. Epidemiology. 1992;3(1):32-9. Epub 1992/01/01. PubMed PMID: 1554808.

64. California Department of Food and Agriculture. Notice of Treatment for the Mediterranean Fruit Fly. Official Notice for the City of Half Moon Bay. Sacramento, CA: State of California; 2017.

65. Plant Health and Pest Prevention Services. Mediterranean Fruit Fly Preventive Release Program CA.gov California Department of Food and Agriculture website; 2014 [updated 2014 Aug 25; cited June 27, 2018]. Available from: <https://www.cdfa.ca.gov/plant/PDEP/prpinfo/>

66. Tsai RJ, Sievert J, Prado J, Buhl K, Stone DL, Forrester M, et al. Acute illness associated with use of pest strips - seven U.S. States and Canada, 2000-2013. MMWR Morb Mortal Wkly Rep. 2014;63(2):42-3. Epub 2014/01/17. PubMed PMID: 24430101; PubMed Central PMCID: PMC4584652.

**Fig.1 Promedio anual de toneladas de plaguicidas OF utilizados en la agricultura, por país, 2010-2015.** El sombreado más oscuro indica un mayor uso. El sombreado de color gris indica que no hubo datos disponibles durante ese período de tiempo. Para los países con datos disponibles para algunos pero no todos los años 2010-2015, se utilizaron los datos disponibles dentro de ese período. La fuente para los datos de los EE. UU. fue [6]; y para todos los demás países, [5]. Mapa creado con mapchart.net. OF, organofosforado

**Fig.S1 Toneladas anuales promedio de plaguicidas OF utilizados en la agricultura, ​​por 1.000 kilómetros cuadrados, por país, 2010–2015.** El sombreado más oscuro indica un mayor uso por 1.000 km cuadrados. El sombreado de color gris indica que no hubo datos disponibles durante ese período de tiempo. Para los países con datos disponibles para algunos pero no todos los años durante 2010-2015, se utilizaron los datos disponibles dentro de ese período. La fuente para los datos de los EE. UU. fue [6]; y para todos los demás países, [5]. Mapa creado con mapchart.net. OF, organofosforado.
