# Supplementary material for: Organophosphate exposures during pregnancy and child neurodevelopment: Recommendations for essential policy reforms
Source: PLoS Med. 2018 Oct 24;15(10):e1002671. doi: 10.1371/journal.pmed.1002671 (PMC6200179; doi:10.1371/journal.pmed.1002671)
Supplement: S2 Text — (DOCX) [file pmed.1002671.s003.docx]

**怀孕期间有机磷农药暴露对儿童神经发育的影响：对基本政策改革的建议**

作者：

Irva Hertz-Picciotto^1*^, Jennifer B. Sass^2^, Stephanie Engel^3^, Deborah H, Bennett^1^, Asa Bradman^4^, Brenda Eskenazi^4^, Bruce Lanphear^5^, Robin Whyatt^6^.

所属机构：

1加州大学戴维斯分校医学院环境健康科学中心和公共卫生科学系

2自然资源保护委员会和华盛顿特区乔治华盛顿大学。

3北卡罗来纳大学教堂山分校流行病学系

4加州大学伯克利分校公共卫生学院

5加拿大不列颠哥伦比亚省温哥华市，西蒙弗雷泽大学，健康科学学院，不列颠哥伦比亚儿童医院

6纽约州哥伦比亚大学Mailman公共卫生学院和儿童环境健康中心

**简称：有机磷农药和神经发育：综述和建议**

**要点：**

•广泛使用有机磷农药来控制昆虫导致人群广泛暴露。

•高浓度的有机磷农药暴露导致中毒和死亡，尤其在发展中国家。

•有充分的证据表明，产前暴露于有机磷农药会使儿童面临认知和行为缺陷以及神经发育障碍的风险

为了保护全球儿童，我们建议：

•政府逐步淘汰毒死蜱和其他有机磷农药;监测水体和其他人类暴露的来源;通过农业生态学的激励和培训促进综合虫害管理（IPM）的使用;并实施与农药有关的疾病的强制性监测。

•健康专业在护理学院和医学院以及继续医学教育课程中添加有机磷农药的危害课程;并教育病人和公众了解这些危害。

•农业实体通过IPM加速开发无毒的害虫防治方法; 在使用有毒化学品时，通过培训和提供防护设备确保工人的安全。

**介绍**

有机磷（Organophosphate）化合物最初是在20世纪30年代至40年代期间作为人类神经毒气剂开发的，有些后来被改制为较低剂量的杀虫剂[1]。高暴露于有机磷化合物会导致乙酰胆碱酯酶不可逆抑制的急性中毒，导致胆碱能综合症（包括瞳孔缩小，唾液分泌增加，支气管收缩，精神恍惚，抽搐或颤抖，并且在有些情况下会导致死亡）。另外，延迟性多发性神经病变也被记载[1]。

在美国，许多有机磷杀虫剂 - 包括马拉硫磷，敌敌畏，谷硫磷和毒死蜱 - 在建立评估人类毒性或生态影响的要求之前被许可用于杀虫剂[2]。由于有机磷杀虫剂在环境中迅速降解，因此被认为比DDT，艾氏剂和狄氏剂等持久性有机氯杀虫剂更安全。 但现在美国环境保护局【3】和/或世卫组织粮食及农业组织【4】认为有40多种有机磷杀虫剂，包括最常用的杀虫剂，对人类健康有中度或高度危害。

最近使用农药的最全面的全球数据库包括五个地区和71个国家报告的信息[5]， 2010 - 2015年农业有机磷农药的年平均用量为13个非洲国家使用了1,145吨; 11个加勒比和中美洲国家使用了4,342吨; 24个欧洲国家使用了10,013吨; 6个南美洲国家使用了13,404吨; 17个亚洲国家使用了29,554吨，印度占主导地位。另外，我们还从美国[6]获得了数据，并绘制了按国家划分的年度有机磷农药使用总量（图1）和每1000平方公里的国家年度总使用量（补充图1）。 有机磷农药在家庭，公园，学校和医院中的广泛使用， 以及在高尔夫球场，通行地带和其他公共场所的使用，已经导致人类有机磷农药暴露无处不在。

有机磷酸盐农药引发一系列健康危害。在这里，我们回顾了有机磷农药对儿童神经发育影响的科学证据。此外，我们还讨论了当前有机磷农药法规的不足之处，并提出了急需的政策改变建议。

**有机磷农药对神经发育的影响**

在美国和其他国家进行的系统综述和多项流行病学研究，涵盖了城市和农业环境中的不同人群，将胎儿发育过程中的有机磷农药暴露与儿童的认知下降，行为和社会发展联系起来[7-11]。通常，这些研究中的有机磷酸盐暴露水平低至不能诱导成人中可测量的胆碱酯酶抑制。在一篇综述中，除了所评估的27项研究中的一项外，其他所有研究都发现有机磷农药暴露对神经发育的不良影响； 产前暴露与神经发育不良之间的关联最为强烈[9]。 胚胎期有机磷农药暴露的结果包括新生儿的原始反射异常；学龄前儿童的精神和运动迟缓；小学年龄儿童的工作和视觉记忆力，处理速度，语言理解，感知推理和智商降低。产前暴露也增加了ADHD和自闭症（ASD）症状或诊断的风险。

与人类研究报告的广泛结果一致，早期有机磷农药对神经发育结局的毒性已在实验动物研究中得到证实。与流行病学研究结果一致，生命早期有机磷农药暴露对认知，行动和社会行为的影响在啮齿动物身上得到了反复证实，而有机磷农药的的浓度只引发少量到几乎不会抑制大脑中的AChE [10,12]。暴露于有机磷农药的时间在受影响的生物化学和解剖学目标以及诱发的特定行为和发育变化中起关键作用[12]。

自流行病学综述发表以来，居住在距离施用有机磷农药的农田1.5公里内的女性所生的孩子被发现ASD诊断的可能性高于超过1.5公里居住的女性;与毒死蜱的关联最强[13]。最近的另一项研究表明，妊娠期母亲尿液中较高的有机磷农药代谢物浓度与青春期发现的ASD特征有关[14]。其他研究小组报告说，在胎儿发育期间，住处邻近施用有机磷农药的农田与7岁时儿童智商降低有关[15]，并且在脐带血中有高浓度的毒死蜱与儿童在大约11岁时轻中度震颤有关 [16]。更大神经发育受损风险见于农场工人的孩子的，因为他们的暴露程度更高[17]，和遗传易感的儿童因为他们对有机磷杀虫剂的解毒能力弱[7]。在调查ASD的同一项研究中，中度至严重的发育迟缓与接近氨基甲酸酯类药物相关，它类似于有机磷农药，但并非有机磷农药[13]。另外两项研究在具有较高社会和经济地位的城市群体中进行，未发现有机磷农药代谢物与智力测验的分数相关[18,19]。尽管如此，证据的权重清楚地表明，在产前发育期间有机磷农药暴露可能对大脑功能有害。

准确测量暴露量对于环境健康研究至关重要。 有机磷农药研究确定暴露有多种方式，包括怀孕期间采集的母亲尿液中有机磷代谢物的定量和脐带血中毒死蜱的直接测量，通过地理上将住宅地址与加州商业杀虫剂应用数据库相关联来量化附近的农药使用量[ 20,21]。加州农药使用报告数据库包含具体的农药数量以及每种应用的日期和地点，已通过两项暴露评估研究进行了验证，该研究表明，在几天到一周内应用的数量与测量的附近环境空气浓度高度相关[22,23]。在所审阅的绝大多数研究中，客观测量（生物学标记和经过验证的农药施用数据）是根据科学建立的方案生成，并且独立于儿童结果而获得。

**对于有机磷酸盐含量低和高的关注**

理解对早期儿童神经发育的影响的关键是高水平暴露后的急性效应与慢性低水平暴露后遗症之间的区别。如上所述，通过抑制酶乙酰胆碱酯酶，高水平的有机磷农药在人类中引起急性的，在某些情况下是致命的影响[2]。实际上，在国际上，农药中毒每年造成200,000人死亡[24]，其中大约99％发生在发展中国家[25]。全球每年约有110,000例农药自身中毒死亡，占报告国家平均死亡人数的13.7％[26]，范围从低收入和中等收入欧洲国家的0.9％到西太平洋区域的低收入和中等收入国家的48.3％。

大量高危害有机磷农药进口到发展中国家。例如，有机磷农药在进口到中美洲国家的24个农药化学品组中排名第四[27]，其中以最大数量进口的两种有机磷农药（terbufos和甲胺磷）已成为“鹿特丹公约”逐步淘汰的目标。该公约是旨在保护人类健康和环境的危险化学品国际贸易协定[4,28]。被农药中毒影响的农场工人往往很少或根本没有接受过使用有害物质的指导，没有提供个人防护设备，和/或使用未妥善维护的应用设备。此外，过度使用，误用和事故导致学童死亡，例如2013年的印度，2014年的中国和2015年的孟加拉国，因为食用含有大量有机磷农药的食物[4,24,29,30] ]。

与这些急性中毒一样悲惨的是，在没有明显中毒的情况下接触有机磷酸盐农药并不意味着没有发生神经系统损害 - 无论是儿童还是成人[31]。美国环境保护署（EPA）在2016年得出结论，现有的流行病学文献提供了“有足够证据表明毒死蜱暴露水平低于能引起乙酰胆碱酯酶抑制所需的神经发育效应”[11]。这种慢性低水平暴露经常被忽视或被视为良性，因为孕妇和胎儿都没有出现临床上明显的体征或症状。此外，发育不良直到数月或数年后才出现。事实上，在科学达到共识的是AChE抑制并不是影响儿童神经发育的前提，慢性低水平暴露的毒性作用发生浓度太低也不能抑制胆碱酯酶[1,9]。因此，证据表明有机磷杀虫剂可以在以前认为安全或无关紧要的水平上干扰大脑发育。

因此，乙酰胆碱酯酶抑制不能用作鉴定对神经发育有害的有机磷农药暴露的生物标志。依赖于乙酰胆碱酯酶抑制作为监管指标掩盖了有机磷农药对早期大脑发育的严重威胁，并且代表了一种不科学且不充分的健康风险评估方法。事实上，其他机制可能介导有机磷对神经系统的毒性导致儿童行为和认知缺陷。毒理学证据表明有机磷农药影响神经炎症，蛋白激酶C受体信号传导，胰岛素抵抗，多巴胺能和谷氨酸能神经传递，以及干扰DNA合成和核转录因子功能。这些机制与大脑发育密切相关[12,32-34]。

实际上,未被发现的危害可能在生命早期暴露的人群中进一步显现出来。胚胎期暴露的影响可能持续到童年中后期。一项研究在年龄2,3,5,7岁发现记忆力损害，智商下降,和注意力不集中或多动症的情况，而另一项研究表明精神发育和推理缺陷在婴儿期和6 - 9岁中（综述[8]）。脐带血有不同浓度毒死蜱的孩子在关于注意力,接受性语言处理、社会认知和调节抑制的脑容量有差异[35]。这些神经解剖上的改变可能形成从农药接触到相关的行为和认知障碍通路上的环节,并永久存在。

**农药监管**

全球农药法规差异很大。与农药使用情况一样，没有数据库为所有国家整合此信息。表1显示了一个或多个国家禁用的47种有机磷杀虫剂[36]的现有数据，以及健康危害程度和禁用每种有机磷杀虫剂的国家数量。目前政府对农药管制的最全面的数据库提供的数据涵盖了美国以外的106个国家获得的47种有机磷杀虫剂中的39种[37]。此数据库中包括完全禁令以及拒绝批准，但不包括限制使用。在106个国家中，81％的国家对39种有机磷杀虫剂中的一种或多种进行了调节[37]。欧盟的28个国家对大多数有机磷农药采取了行动（33）。其他已禁止10个以上的国家包括美国（26），柬埔寨（15），中国（15），沙特阿拉伯（15），几内亚（12），韩国（12）毛里塔尼亚（12）和泰国（12）。值得注意的是，制定法规并不一定意味着它们得到执行。此外，在几十个国家禁用的一些毒性最大的农药杀虫剂出口到其他地方，往往出口到发展中国家，有时大量出口，例如到哥斯达黎加和危地马拉[27]。在墨西哥，使用至少十二种被世界卫生组织和世界粮农组织列为高度危险的有机磷杀虫剂[38]。

| 表1:  有机磷杀虫剂，危害程度以及禁止它们的国家数量 | | | | | | |
| --- | --- | --- | --- | --- | --- | --- |
|  | 化合物^3^ | 危害等级：  E =非常危险  H =高度危险  M =中度危险  S =有点危险  ‘--'未分类H由PAN'  ‘**’ 未分类 | | | 禁用的国家数 | 在美国禁用的用X表示。所有其它所列有机磷农药， 现都在美国注册使用 |
|  |  | U.S. EPA^4^ | FAO-WHO^5^ | PAN^6^ |  |  |
| 1 | 乙酰甲胺磷 | M | M | H | 31 |  |
| 2 | 谷硫磷 | H | H | H | 39 | X |
| 3 | 硫线磷 | ** | H | H | 31 |  |
| 4 | 四氯乙磷 | ** | E | H | 29 |  |
| 5 | 毒虫畏 | H | H | H | 35 | X |
| 6 | 毒死蜱 | M | M | H | 2 |  |
| 7 | 甲基毒死蜱 | ** | S | H | 1 |  |
| 8 | 虫螨磷^,7,8^ | H | ** | ** | ** | X |
| 9 | 蝇毒磷 | H | H | H | 30 |  |
| 10 | 敌敌畏 | M | H | H | 32 |  |
| 11 | 氯亚磷/氯亚胺硫磷^7,8^ | H | ** | ** | ** | X |
| 12 | 二嗪磷 | M | M | H | 30 |  |
| 13 | 百治磷 | H | H | H | 34 |  |
| 14 | 乐果 | ** | M | H | 4 |  |
| 15 | 敌杀磷^7,8^ | H | ** | ** | ** | X |
| 16 | 二硫 | H | E | H | 38 | X |
| 17 | 乙硫磷 | M | M | -- | 30 | X |
| 18 | 丙线磷（灭线磷） | M | E | H | 8 |  |
| 19 | 乙基对硫磷^8^ | H | ** | ** | ** | X |
| 20 | 苯线磷 | H | H | H | 6 | X |
| 21 | 杀螟松 | M | M | H | 28 |  |
| 22 | 倍硫磷 | M | M | H | 30 | X |
| 23 | 地虫磷 (fenophos) ^7^ | H | ** | -- | 33 | X |
| 24 | 氯唑磷^7,8^ | ** | ** | ** | ** | X |
| 25 | 异柳磷^7^ | H | ** | -- | 29 | X |
| 26 | 马拉硫磷 | M | S | H | 2 |  |
| 27 | 甲胺磷 | H | H | H | 49 | X |
| 28 | 杀扑磷 | H | H | H | 34 | X |
| 29 | 甲基对硫磷 | H | E | H | 59 | X |
| 30 | 速灭磷 | H | E | H | 37 | X |
| 31 | 久效磷 | H | H | H | 60 | X |
| 32 | 二溴磷 | M | M | H | 28 |  |
| 33 | 砜吸磷甲基 | M | H | H | 30 | X |
| 34 | 甲拌磷 | H | E | H | 37 |  |
| 35 | 伏杀硫磷 | M | M | -- | 29 | X |
| 36 | 磷胺 ^8^ | M | M | ** | ** |  |
| 37 | 磷胺 | H | E | H | 49 | X |
| 38 | 丁基嘧啶磷^8^ | ** | ** | ** | ** |  |
| 39 | 甲基嘧啶磷^8^ | M | M | ** | ** |  |
| 40 | 丙溴磷 | M | M | H | 29 | X |
| 41 | 强敌 | M | H | H | 28 | X |
| 42 | 治螟 | H | E | H | 32 | X |
| 43 | 硫丙磷^7,8^ | M | ** | ** | ** | X |
| 44 | 双硫磷 | M | S | H | 28 | X |
| 45 | 特丁硫磷 | H | E | H | 34 |  |
| 46 | 畏 | M | ** | H | 28 |  |
| 47 | 敌百虫 | M | M | H | 32 |  |
| ^1^该有机磷杀虫剂清单来自美国环保局农药计划办公室“有机磷累积风险评估，2006年更新”[36]（表ES-1，第16页“2006年累积风险评估更新中考虑的有机磷农药”）我们从中排除了那些不是杀昆虫剂的杀虫剂。  ^2^来自PAN国际禁用农药综合清单[37]  （http://pan-international.org/pan-international-consolidated-list-of-banned-pesticides/）。收集这些数据的方法和来源在解释性说明中描述：（http://pan-international.org/wp-content/uploads/Consolidated-List-of-Bans-Explanatory-2017April.pdf）。此列表不包括限制，仅限禁止或不批准的决定。.  ^3^美国的“禁用”农药被定义为一种农药，所有注册用途都被最终的环保署采取行动所禁止，并包括通过工业界和美国环保署之间的自愿协议撤销的农药。 JBS与美国环境保护局农药计划办公室农药再评价处处长Yu-Ting Guilaran于7月12日13日和 23日签署的书面通讯中提供了在美国禁用或注册使用的有机磷的状况。 2018年2月23日。  ^4^危害等级：美国环境保护局，农药计划办公室。农药中毒的认识与管理，第六版。 Roberts J和Reigart J，第5章。有机磷 [3]。华盛顿特区，2013年。网址：http：//www2.epa.gov/pesticide-worker-safety。 “H”表示高度危险，“M”表示中度危险。  ^5^危害等级：粮食及农业组织 - 世界卫生组织，“世界卫生组织推荐的危害农药分类”（最后一次访问时间为2018年9月1日）（http://www.who.int/ipcs/publications/pesticides_hazard_2009.pdf?ua = 1）。 “高度危险农药”的概念和标准最初在其2008年第2次报告“粮农组织/世界卫生组织第二届农药管理联席会议报告”（最后一次更新于2018年7月）中进行了描述（http://www.fao.org /fileadmin/templates/agphome/documents/Pests_Pesticides/Code/Report.pdf）。随着对农药毒性机制的科学理解的推进，这些已被纳入，如2016年出版的“国际农药管理高危农药行为准则”中所述（http://apps.who.int/iris/bitstream/handle/10665/205561/9789241510417_eng.pdf;jsessionid=D3B3CCA5B28692A5F3D437B2CF7F0AA0?sequence=1).）。      联合国粮农组织 - 世界卫生组织JMPM将禁用的农药定义为：“禁用的农药是指最终管制行动禁止使用的农药，以保护人类健康或环境。它包括首次审核就被拒绝批准使用的农药，或者已被国内市场或国内批准程序中的进一步考虑中从行业撤回的农药，并且有明确证据表明此类行动已被采取的顺序保护人类健康或环境。“    ^6^  危害等级：农药行动网络，禁用农药国际综合清单，2017年4月  ^7^根据世界卫生组织推荐的农药危害分类，2010年被认为已过时或不再用作农药。  ^8^不包含在PAN数据库中 | | | | | | |

在美国，EPA根据两项重叠法规管制农药，即联邦食品，药品和化妆品法案（FFDCA）和联邦杀虫剂，杀菌剂和灭鼠剂法案（FIFRA）。许多在美国被禁用的杀虫剂最初是在1970年之前获得许可的，当时所需的健康和安全评估很少，并且在美国环保局成立之前。由于19世纪70年代的立法要求加强健康和安全研究，制造商和EPA之间达成了自愿协议，取消或逐步淘汰某些农药的注册，包括18种有机磷杀虫剂。

1996年，“食品质量保护法”（FQPA）修订了FIFRA和FFDCA，要求EPA包含额外的安全因素以保护儿童免于更多的暴露于有机磷酸盐环境和增高他们的易感性[39]。由于每单位体重摄入的食物，水和空气儿童比成人多，从而儿童的农药负担较大; 他们通过口腔行为探索世界; 他们经常在农药和其他有毒化学物质沉淀的地板上爬行或玩耍。早期易感性增加的部分原因是未成熟的解毒的酶系统，包括PON1 [7,40,41]。根据FQPA，EPA必须证明有合理的理由确定不会因总体暴露在农药的含量而造成伤害，包括所有预期的膳食暴露和所有其他有可靠来源的暴露。

在FQPA通过后，所有市场部门的有机磷杀虫剂使用量下降了70％以上，从2000年的每年7000万磅（lbs / yr）到2012年的约2000万磅/年（最新的可用数据）[6] 。到2002年，根据美国环保署和杀虫剂生产商之间达成的协议，大多数非农业用途被淘汰，基于美国环保署对毒死蜱和二嗪农的风险评估结果显示居民特别是儿童的来自于住宅害虫控制的风险高得令人无法接受[42,43] 。 1994年至2004年间，儿童通常食用的食品中（例如，水果）使用的有机磷杀虫剂的数量减少了57％，每年使用的活性成分为28至1200万磅（12,701至5,443公吨）[44]。这一举措导致美国人口血液和尿液中有机磷浓度显着降低[45]。然而，农业有机磷农药的使用继续导致农场工人及其家庭[15]和居家人口，学校儿童和农田附近的其他人[23]的暴露，以及影响更广泛人口的食物和饮用水污染。

2016年，美国环保署得出结论认为，单独通过食物或饮用水接触毒死蜱（美国最常用的有机磷酸盐杀虫剂）可能会导致人口暴露程度高得令人无法接受，并确定一些生育年龄的妇女，婴儿和儿童摄入的毒死蜱水平大大高于这些脆弱生命阶段的可接受水平[11]。美国环保署还发现了许多可能导致农场工人和其他在旁边的人不安全暴露的情景。出于这些原因，根据法律要求，EPA提议撤销允许食品中毒死蜱残留的所有标准（称为耐受量）。撤销这些容忍措施基本上会禁止粮食作物的有机磷农药 [11]。然而，在2017年3月，尽管有大量的毒性证据，并且与EPA自己的风险评估相反，特朗普政府EPA宣布“ 解释神经发育影响的科学仍然没有得到解决，并且有必要进一步评估该科学...... [因此]有必要对目前人类暴露于毒死蜱的过程中是否存在发生不良神经发育影响的潜在可能性进行进一步确定” ，认为该机构不能取消对毒死蜱的任何使用[46]。此行动将推迟可能的监管行动，直到2022年10月。然而，在2018年8月9日，美国法院于第9巡回命令美国EPA在60天内完成对毒死蜱的禁令，包括禁止在美国销售和禁止被杀虫剂污染的食品进入美国市场。法院根据EPA 2016年的调查结果确定农药不符合联邦安全标准，特别是对婴儿和儿童有害。在2018年9月，EPA提交了一份情愿书，要求重新审核关于毒死蜱的法案。

**建议：**

2014年，美国儿科学会呼吁儿科医生和政府通过教育，农药标签，公共卫生监督和监管行动来识别和减少农药接触[47]。 2016年，一个独立的科学家和健康专业人员小组发布了“TENDR项目共识声明”，作为国家行动呼吁，以期大幅减少化学品暴露，包括有机磷杀虫剂，因为这些化学物质被认定为将增加美国儿童，并可能是整个世界的儿童，神经发育障碍的风险[48]。 TENDR项目得出结论认为，有机磷农药暴露对儿童神经发育的重大风险的证据表示我们需要采取强有力的监管措施。 2017年，联合国关于食物权的报告呼吁改变农业做法，以确保食品安全，无农药，质量充足[24]。为了实现减少有机磷杀虫剂暴露的目标，我们因此为政府，公共卫生和医疗机构或组织以及农业实体提出了一项行动计划。我们的建议在下面详细说明。这些步骤将显著减少产前和儿童接触有机磷农药的风险。

**我们建议政府采取以下行动：**

•全球国家和州或省政府：逐步淘汰农业中所有有机磷农药;

•全球国家和州或省政府：禁止非农业使用所有有机磷农药，包括家用产品;

•美国环保署：取消毒死蜱的所有食品耐受量，正如该机构在前文中提出;

•美国环保署和州政府：逐步淘汰农业中所有其他有机磷农药的使用;

•美国环保署：禁止使用少数剩余的有机磷农药进行非农业害虫防治;

•在此期间，国家，州和地方机构：采取措施减少人体暴露（例如，在施用有机磷农药之前要求提前通知附近居民和学校; 通过对空中喷洒和空气喷射等应用方法实施限制以减少漂流暴露并保护水和敏感场所，如家庭和学校）;

•国家，州和地方机构：定期监测流域，确保有机磷农药不会继续污染湖泊，河流和溪流，包括那些饮用水源，并实施有针对性的饮用水监测;

•国家和州机构：在全国范围内或通过全州协调计划建立有效的农药综合使用和疾病报告计划。

我们建议医学院，公共卫生项目和医疗保健协会：

•组织持续医学教育课程，向医疗保健提供者提供有毒化学品暴露的急性和慢性影响的教育，包括：如何识别和治疗接受高有机磷暴露的儿童;如何建议孕妇和幼儿的父母采取措施避免从虱子，跳蚤和蜱虫身上接触到农药[49]，以及从草坪和园艺产品，附近农田，高尔夫球场，学校和商场等处接触到; 从水果和蔬菜中清除潜在农药残留的适当方法，以及含农药量最高的农产品。

•教育医疗服务提供者如何向国家监测部门提供必要的农药中毒报告。

•鼓励护理学院和医学院提供包括杀虫剂在内的环境危害课程，并在其考试中纳入环境健康。

我们建议农业实体：

•以最适当的语言和相关的教育水平，为农民在处理和应用以及工人保护标准方面提供更好的培训。在美国，这意味着EPA工人保护标准机构需要定期进行培训

•教育工人如何避免将农药暴露带回家;

•制定环境友好型防治害虫的方法 - 综合虫害管理（IPM） - 目标是消除或减少食物中有毒化学物质。

各种政府层面已经采取了相关的示例性行动。欧盟选择不批准接近200种农药，其中20多种是有机磷农药，并且多个国家已经对敌敌畏，甲胺磷和甲基对硫磷等有机磷农药实施禁令[37]。在美国，加利福尼亚已采取措施限制儿童在学校和儿童保育设施附近农药的使用[50]，夏威夷最近禁止分发，销售，运输和使用任何含有毒死蜱作为活性成分的农药[51]。

在减少有机磷杀虫剂的使用中，替代或替代化学品的毒性作用需要仔细检查。拟除虫菊酯农药已取代有机磷作为住宅害虫防治产品中的主要杀虫剂类别，但最近的啮齿动物实验室研究和流行病学研究表明，产前的拟除虫菊酯农药暴露也可能增加不良神经发育和行为及负面情绪的风险[13,52-54] 。新烟碱类杀虫剂现在是美国作物中增长最快的一类杀虫剂[55];它们在植物，土壤和水中持久存在，对无脊椎动物（包括濒临灭绝的水生物种，蜜蜂和其他有益昆虫）具有高毒性[56]。此外，广泛和系统性农药使用的影响已被充分证明，对陆地，水生，湿地，海洋和底栖生境造成了严重的负面生态后果，并对生态系统功能和恢复力构成风险。

如果除有机磷酸盐之外的合成杀虫剂也具有神经毒性，有哪些替代品？

**农业：**有机磷酸盐农药的绝大部分用于农作物和畜牧业。需要广泛实施综合虫害管理（IPM）以减少这种使用。 IPM是一种降低风险的病虫害管理策略，强调检查，监测，预防和害虫控制，使用最少毒性的方法，包括（农业）文化习俗，如间作（在近距离种植两种或更多种作物，这可以减少对疾病和害虫），作物轮作和覆盖作物（以减少土壤侵蚀和改善土壤健康），物理控制如陷阱或虫子真空吸尘器，鼓励有益昆虫的栖息地管理，以及生物控制，如寄生蜂的释放控制蚜虫，农药只作为最后时期的必要手段使用。必须使用时，首先选择毒性最小的农药，例如批准用于有机农业的材料（例如，苏云金芽孢杆菌控制鳞翅目）[57]。

虽然IPM策略原则上不禁止使用有机磷和其他神经毒性农药，但这些高风险材料是最后的手段，应以保护人类和环境健康的方式应用。有足够的证据表明，大多数施用有机磷杀虫剂的作物也是可能有机生产的，从而证明了有机磷杀虫剂不是必需的[58]。一些顽固害虫可能难以用较低毒性的农药来管理，这在某些情况下可能导致较低的产量或较高的生产成本，从而降低竞争力。然而，最近的研究表明，有机和其他替代生产系统的作物产量正在增加，并且在某些情况下与常规产量相匹配[59];这些方法还可能降低公共健康和环境的外部成本[60]。为了确保农民不会因成本上升和利润空间减少而受到威胁，许多农业贸易和政策组织建议政府增加对扩展研究的支持，以及支持向低毒性材料过渡所需的外展[61]。

**公共卫生**：有机磷杀虫剂用于蚊子和其他病媒控制，以防止媒介传播的疾病，如寨卡病毒或西尼罗河病毒。我们不建议有害生物管理的突然变化，这样会增加接触这些病毒的风险。我们确实提倡增加资金，以便更好地了解这些和其他载体的生态学和生物学及其传播的疾病，以及在不使用有机磷或其他神经毒性农药的情况下控制它们的替代方法。加利福尼亚地中海果蝇是一个很好的历史实例， 地中海果蝇是一种严重的侵入性农业害虫，为疾病媒介的应用提供了一个传播媒介。在20世纪90年代早期，国家官员使用直升机在住宅区喷洒马拉硫磷，超过200万人居住在该区域[62]。随后的关注[63]导致开发了一种全面的无菌果蝇释放计划，该计划与经常使用有机认可的农药的局部处理相结合，成功地控制了虫害，而不需要在广阔的居住区域使用有机磷酸盐农药[64,65] 。对于新的入侵物种也应该考虑类似的策略，例如斑点灯笼蝇，目前威胁美国东部的生态系统和农业。综合虫害管理倾向于使用毒性最小的选项。

**结构，室内和景观的害虫控制：**室内和景观农药应用可导致高度暴露。 Dichlorvos是一种已在许多国家被禁用的有机磷农药，美国政府仍就允许在室内使用杀飞虫。同样，马拉硫磷仍然出售用于景观和花园。由于慢性，低水平暴露导致健康不良的风险，以及据报道美国消费者急性中毒[66]，我们建议立即淘汰有机磷农药的所有室内和景观使用，特别是在儿童活动的环境中。应在这些环境中应用基本的IPM原则，包括有害生物排除（即屏幕）和陷阱。

为了保持健康和可持续性，室内和室外害虫管理必须最终依赖于无毒或毒性较小的替代品;同时，农业需要更强有力的支持，以采用系统方法，最大限度地减少神经毒性农药的使用，同时为农民提供健康食品和经济可持续性。特别报告员向联合国大会提交的食物权报告阐述了一个类似的理念：为了成功减少或消除危险农药的使用，国际社会的努力将需要解决目前嵌入在农业政策方面的生态，社会和经济因素方面的问题。在国家层面，这将需要具有挑战性的依赖农业化学将农业进行重组并寻求最安全的可行替代方案[24]。我们加入美国儿科学会和联合国，建议密切监测农药中毒，激励非化学方法控制虫害，监测含有农药的水和食物来源，并通过全面披露，标识和进一步公布农药配方等加强公众的知情权。最后，我们认为，向公众广泛传播有关农药暴露的来源及其对健康的不利影响，是文明社会，医学界和农业产业的道德和社会责任，还须制定农业生态学培训计划，以实现粮食生产的范式转变。

致谢： 我们感谢Maureen Swanson的支持。

**参考文献:**

1. Costa LG. Organophosphorus Compounds at 80: Some Old and New Issues. Toxicol Sci. 2018;162(1):24-35. Epub 2017/12/12. doi: 10.1093/toxsci/kfx266. PubMed PMID: 29228398.

2. Soltaninejad K S, S. History of the Use and Epidemiology of Organophosphorus Poisoning. In: Basic and Clinical Toxicology of Organophosphorus Compounds Editors: Mahdi Balali-Mood, Mohammad Abdollahi, Springer. 2014.

3. Roberts J, Reigart J. Chapter 5. Organophosphates. Recognition and Management of Pesticide Poisonings. Sixth ed. Washington, D.C.: U.S. ENvironmental Protection Agency, Office of Pesticide Programs; 2013.

4. World Health Organization International Programme on Chemical Safety. The WHO Recommended Classification of Pesticides by Hazard and Guidelines to Classification 2009. Geneva, Switzerland: 2009.

5. Pesticides Use Data [Internet]. 2017 [cited June 28, 2018]. Available from: <http://www.fao.org/faostat/en/#data/RP>.

6. U.S. EPA. Report of Pesticide Industry Sales and Usage, 2008-2012 Market Estimates. United States Environmental Protection Agency, Washington, DC [cited July 11, 2018] <https://wwwepagov/pesticides/pesticides-industry-sales-and-usage-2008-2012-market-estimates>. 2017.

7. Gonzalez-Alzaga B, Lacasana M, Aguilar-Garduno C, Rodriguez-Barranco M, Ballester F, Rebagliato M, et al. A systematic review of neurodevelopmental effects of prenatal and postnatal organophosphate pesticide exposure. Toxicol Lett. 2014;230(2):104-21. doi: 10.1016/j.toxlet.2013.11.019. PubMed PMID: 24291036.

8. Koureas M, Tsakalof A, Tsatsakis A, Hadjichristodoulou C. Systematic review of biomonitoring studies to determine the association between exposure to organophosphorus and pyrethroid insecticides and human health outcomes. Toxicol Lett. 2012;210(2):155-68. doi: 10.1016/j.toxlet.2011.10.007. PubMed PMID: 22020228.

9. Munoz-Quezada MT, Lucero BA, Barr DB, Steenland K, Levy K, Ryan PB, et al. Neurodevelopmental effects in children associated with exposure to organophosphate pesticides: a systematic review. Neurotoxicology. 2013;39:158-68. doi: 10.1016/j.neuro.2013.09.003. PubMed PMID: 24121005; PubMed Central PMCID: PMCPMC3899350.

10. U.S. EPA. EPA Revised Human Health Risk Assessment on Chlorpyrifos. December 2014. Docket ID EPA-HQ-OPP-2008-0850 <http://www.epa.gov/ingredients-used-pesticide-products/revised-human-health-risk-assessment-chlorpyrifos>. 2014.

11. U.S. EPA. Chlorpyrifos: Revised Human Health Risk Assessment for Registration Review Document ID: EPA-HQ-2015-0653-0454 <https://www.regulations.gov/document?D=EPA-HQ-OPP-2015-0653-0454>. US Environmental Protection Agency Washington, DC 20460. 2016.

12. Abreu-Villaca Y, Levin ED. Developmental neurotoxicity of succeeding generations of insecticides. Environ Int. 2017;99:55-77. Epub 2016/12/03. doi: 10.1016/j.envint.2016.11.019. PubMed PMID: 27908457; PubMed Central PMCID: PMCPMC5285268.

13. Shelton JF, Geraghty EM, Tancredi DJ, Delwiche LD, Schmidt RJ, Ritz B, et al. Neurodevelopmental disorders and prenatal residential proximity to agricultural pesticides: the CHARGE study. Environ Health Perspect. 2014;122(10):1103-9. doi: 10.1289/ehp.1307044. PubMed PMID: 24954055; PubMed Central PMCID: PMCPMC4181917.

14. Sagiv SK, Harris MH, Gunier RB, Kogut KR, Harley KG, Deardorff J, et al. Prenatal Organophosphate Pesticide Exposure and Traits Related to Autism Spectrum Disorders in a Population Living in Proximity to Agriculture. Environ Health Perspect. 2018;126(4):047012. Epub 2018/04/28. doi: 10.1289/EHP2580. PubMed PMID: 29701446.

15. Gunier RB, Bradman A, Harley KG, Kogut K, Eskenazi B. Prenatal Residential Proximity to Agricultural Pesticide Use and IQ in 7-Year-Old Children. Environ Health Perspect. 2017;125(5):057002. doi: 10.1289/EHP504. PubMed PMID: 28557711.

16. Rauh VA, Garcia WE, Whyatt RM, Horton MK, Barr DB, Louis ED. Prenatal exposure to the organophosphate pesticide chlorpyrifos and childhood tremor. Neurotoxicology. 2015;51:80-6. doi: 10.1016/j.neuro.2015.09.004. PubMed PMID: 26385760.

17. Engel SM, Bradman A, Wolff MS, Rauh VA, Harley KG, Yang JH, et al. Prenatal Organophosphorus Pesticide Exposure and Child Neurodevelopment at 24 Months: An Analysis of Four Birth Cohorts. Environ Health Perspect. 2016;124(6):822-30. doi: 10.1289/ehp.1409474. PubMed PMID: 26418669; PubMed Central PMCID: PMCPMC4892910.

18. Cartier C, Warembourg C, Le Maner-Idrissi G, Lacroix A, Rouget F, Monfort C, et al. Organophosphate Insecticide Metabolites in Prenatal and Childhood Urine Samples and Intelligence Scores at 6 Years of Age: Results from the Mother-Child PELAGIE Cohort (France). Environ Health Persp. 2016;124(5):674-80. doi: 10.1289/ehp.1409472. PubMed PMID: WOS:000377077000025.

19. Donauer S, Altaye M, Xu Y, Sucharew H, Succop P, Calafat AM, et al. An Observational Study to Evaluate Associations Between Low-Level Gestational Exposure to Organophosphate Pesticides and Cognition During Early Childhood. Am J Epidemiol. 2016;184(5):410-8. Epub 2016/08/20. doi: 10.1093/aje/kwv447. PubMed PMID: 27539379; PubMed Central PMCID: PMCPMC5013882.

20. California Department of Pesticide Regulation. Pesticide Use Reporting (PUR) (Home Page) [accessed August 2017]. Available from: <https://www.cdpr.ca.gov/docs/pur/purmain.htm>.

21. California Department of Pesticide Regulation. Overview of Pesticide Use Reporting 2000.

22. Harnly M, McLaughlin R, Bradman A, Anderson M, Gunier R. Correlating agricultural use of organophosphates with outdoor air concentrations: a particular concern for children. Environ Health Perspect. 2005;113(9):1184-9. Epub 2005/09/06. PubMed PMID: 16140625; PubMed Central PMCID: PMCPMC1280399.

23. Wofford P, Segawa R, Schreider J, Federighi V, Neal R, Brattesani M. Community air monitoring for pesticides. Part 3: using health-based screening levels to evaluate results collected for a year. Environ Monit Assess. 2014;186(3):1355-70. doi: 10.1007/s10661-013-3394-x. PubMed PMID: 24370859.

24. United Nations. Report of the U.N. Special Rapporteur on the Right to Food. Human Rights Council 34^th^ Session; 2017.

25. Goldman LR. Childhood Pesticide Poisoning: Information for Advocacy and Action. Geneva, Switzerland: United Nations Environment Programme and WHO, 2004.

26. Mew EJ, Padmanathan P, Konradsen F, Eddleston M, Chang SS, Phillips MR, et al. The global burden of fatal self-poisoning with pesticides 2006-15: Systematic review. J Affect Disord. 2017;219:93-104. Epub 2017/05/24. doi: 10.1016/j.jad.2017.05.002. PubMed PMID: 28535450.

27. Bravo V, Rodriguez T, van Wendel de Joode B, Canto N, Calderon GR, Turcios M, et al. Monitoring pesticide use and associated health hazards in Central America. Int J Occup Environ Health. 2011;17(3):258-69. Epub 2011/09/13. doi: 10.1179/107735211799041896. PubMed PMID: 21905395.

28. Rotterdam Convention on the Prior Informed Consent Procedure for Certain Hazardous Chemicals and Pesticides in International Trade, (2004, Revised 2015).

29. Reuters. Pesticide Found in Meals That Killed Indian Children, Official Says. New York Times. 2013.

30. Roy P, Karmakar K. Pesticide in litchi kills kids, again. The Daily Star, . 2015.

31. Starks SE, Hoppin JA, Kamel F, Lynch CF, Jones MP, Alavanja MC, et al. Peripheral nervous system function and organophosphate pesticide use among licensed pesticide applicators in the Agricultural Health Study. Environ Health Perspect. 2012;120(4):515-20. Epub 2012/01/21. doi: 10.1289/ehp.1103944. PubMed PMID: 22262687; PubMed Central PMCID: PMCPMC3339452.

32. Banks CN, Lein PJ. A review of experimental evidence linking neurotoxic organophosphorus compounds and inflammation. Neurotoxicology. 2012;33(3):575-84. doi: 10.1016/j.neuro.2012.02.002. PubMed PMID: 22342984; PubMed Central PMCID: PMCPMC3358519.

33. Bjorling-Poulsen M, Andersen HR, Grandjean P. Potential developmental neurotoxicity of pesticides used in Europe. Environ Health. 2008;7:50. doi: 10.1186/1476-069X-7-50. PubMed PMID: 18945337; PubMed Central PMCID: PMCPMC2577708.

34. Lasram MM, Dhouib IB, Annabi A, El Fazaa S, Gharbi N. A review on the molecular mechanisms involved in insulin resistance induced by organophosphorus pesticides. Toxicology. 2014;322:1-13. doi: 10.1016/j.tox.2014.04.009. PubMed PMID: 24801903.

35. Rauh VA, Perera FP, Horton MK, Whyatt RM, Bansal R, Hao X, et al. Brain anomalies in children exposed prenatally to a common organophosphate pesticide. Proc Natl Acad Sci U S A. 2012;109(20):7871-6. doi: 10.1073/pnas.1203396109. PubMed PMID: 22547821; PubMed Central PMCID: PMCPMC3356641.

36. U.S. EPA Office of Pesticide Programs. Organophosphorus Cumulative Risk Assessment, 2006 Update. 2006. p. 522 (190 without the appendices).

37. Pesticide Action Network. International Consolidated List of Banned Pesticides 2017 [Accessed July 6, 2018] http://pan-international.org/pan-international-consolidated-list-of-banned-pesticides/.

38. Bejarano Gonzales F, editor. Highly Hazardous Pesticides in Mexico. 1st English Edition ed: RAPAM; 2018.

39. U.S. EPA. Food Quality Protection Act (FQPA). 1996.

40. Engel SM, Wetmur J, Chen J, Zhu C, Barr DB, Canfield RL, et al. Prenatal exposure to organophosphates, paraoxonase 1, and cognitive development in childhood. Environ Health Perspect. 2011;119(8):1182-8. doi: 10.1289/ehp.1003183. PubMed PMID: 21507778; PubMed Central PMCID: PMCPMC3237356.

41. Huen K, Harley K, Brooks J, Hubbard A, Bradman A, Eskenazi B, et al. Developmental changes in PON1 enzyme activity in young children and effects of PON1 polymorphisms. Environ Health Perspect. 2009;117(10):1632-8. Epub 2009/12/19. doi: 10.1289/ehp.0900870. PubMed PMID: 20019917; PubMed Central PMCID: PMCPMC2790521.

42. U.S. EPA. Diazinon Revised Risk Assessment and Agreement with Registrants <http://www.oda.state.ok.us/forms/cps/epaagree.pdf>. 2001.

43. U.S. EPA. Chlorpyrifos Revised Risk Assessment and Agreement with Registrants <http://www.ibiblio.org/london/NAFEX/message-archives/old/pdf00000.pdf>. Washington, DC:US Environmental Protection Agency. 2000.

44. Edwards D, U.S. EPA,. Reregistration and Tolerance Reassessment. . U.S. Environmental Protection Agency; 2006.

45. Clune AL, Ryan PB, Barr DB. Have regulatory efforts to reduce organophosphorus insecticide exposures been effective? Environ Health Perspect. 2012;120(4):521-5. doi: 10.1289/ehp.1104323. PubMed PMID: 22251442; PubMed Central PMCID: PMCPMC3339465.

46. U. S. EPA. Federal Register for Wednesday, April 5, 2017 (FR 16581) (FRL–9960–77) EPA–HQ–OPP–2007–1005; Chlorpyrifos; Order Denying PANNA and NRDC’s Petition To Revoke Tolerances. Docket ID EPA-HQ-OPP-2007-1005-0100. Available at: <https://www.regulations.gov/document?D=EPA-HQ-OPP-2007-1005-0100>. US Environmental Protection Agency Washington, DC 20460. 2017.

47. Roberts JR, Karr CJ, Council On Environmental H. Pesticide exposure in children. Pediatrics. 2012;130(6):e1765-88. Epub 2012/11/28. doi: 10.1542/peds.2012-2758. PubMed PMID: 23184105; PubMed Central PMCID: PMCPMC5813803.

48. Bennett D, Bellinger DC, Birnbaum LS, Bradman A, Chen A, Cory-Slechta DA, et al. Project TENDR: Targeting Environmental Neuro-Developmental Risks The TENDR Consensus Statement. Environ Health Perspect. 2016;124(7):A118-22. doi: 10.1289/EHP358. PubMed PMID: 27479987; PubMed Central PMCID: PMCPMC4937840.

49. Centers for Disease Control and Prevention. Head Lice – General Guidelines. <http://www.cdc.gov/parasites/lice/head/treatment.html>. Centers for Disease Control and Prevention Atlanta Georgia. 2016.

50. Regulation to Address Pesticides Used Near Schools and Child Day Care Facilities, California Code of Regulations, Title 3. Sect. Sections 6690-6692 (2018).

51. Reese D. Hawaii Becomes First State to Ban Popular Pesticide2018. Available from: <https://www.courthousenews.com/hawaii-becomes-first-state-to-ban-popular-pesticide/>.

52. Eskenazi B, An S, Rauch SA, Coker ES, Maphula A, Obida M, et al. Prenatal Exposure to DDT and Pyrethroids for Malaria Control and Child Neurodevelopment: The VHEMBE Cohort, South Africa. Environ Health Perspect. 2018;126(4):047004. Epub 2018/04/13. doi: 10.1289/EHP2129. PubMed PMID: 29648420.

53. Furlong MA, Barr DB, Wolff MS, Engel SM. Prenatal exposure to pyrethroid pesticides and childhood behavior and executive functioning. Neurotoxicology. 2017;62:231-8. Epub 2017/08/16. doi: 10.1016/j.neuro.2017.08.005. PubMed PMID: 28811173; PubMed Central PMCID: PMCPMC5623638.

54. Richardson JR, Taylor MM, Shalat SL, Guillot TS, 3rd, Caudle WM, Hossain MM, et al. Developmental pesticide exposure reproduces features of attention deficit hyperactivity disorder. FASEB J. 2015;29(5):1960-72. doi: 10.1096/fj.14-260901. PubMed PMID: 25630971; PubMed Central PMCID: PMCPMC4415012.

55. Jeschke P, Nauen R, Schindler M, Elbert A. Overview of the status and global strategy for neonicotinoids. J Agric Food Chem. 2011;59(7):2897-908. doi: 10.1021/jf101303g. PubMed PMID: 20565065.

56. The Task Force on Systemic Pesticides (TFSP). Worldwide Integrated Assessment of the Impacts of Systemic Pesticides on Biodiversity and Ecosystems. Available at: <http://www.tfsp.info/worldwide-integrated-assessment/>. 2015.

57. Organic Materials Review Institute (OMRI). OMRI Products LIst Eugene, OR [6/27/18]. Available from: <https://www.omri.org/omri-lists>.

58. National Agricultural Statistics Service. Certified Organic Survey 2016 Summary. Washington, D.C. : U.S. Department of Agriculture; 2017.

59. Ponisio LC, M'Gonigle LK, Mace KC, Palomino J, de Valpine P, Kremen C. Diversification practices reduce organic to conventional yield gap. Proc Biol Sci. 2015;282(1799):20141396. Epub 2015/01/27. PubMed PMID: 25621333; PubMed Central PMCID: PMCPMC4286047.

60. Bellinger DC. A strategy for comparing the contributions of environmental chemicals and other risk factors to neurodevelopment of children. Environ Health Perspect. 2012;120(4):501-7. Epub 2011/12/21. doi: 10.1289/ehp.1104170. PubMed PMID: 22182676; PubMed Central PMCID: PMCPMC3339460.

61. Organic Trade Association and The Organic Center applaud bipartisan bill to invest in organic ag research [Internet]. Organic Trade Association; 2017. Available from: <https://www.ota.com/news/press-releases/19672>

62. Bradman MA, Harnly ME, Goldman LR, Marty MA, Dawson SV, Dibartolomeis MJ. Malathion and malaoxon environmental levels used for exposure assessment and risk characterization of aerial applications to residential areas of southern California, 1989-1990. J Expo Anal Environ Epidemiol. 1994;4(1):49-63. Epub 1994/01/01. PubMed PMID: 7894268.

63. Thomas DC, Petitti DB, Goldhaber M, Swan SH, Rappaport EB, Hertz-Picciotto I. Reproductive outcomes in relation to malathion spraying in the San Francisco Bay Area, 1981-1982. Epidemiology. 1992;3(1):32-9. Epub 1992/01/01. PubMed PMID: 1554808.

64. California Department of Food and Agriculture. Notice of Treatment for the Mediterranean Fruit Fly. Official Notice for the City of Half Moon Bay. . Sacramento, CA: State of California; 2017.

65. Plant Health and Pest Prevention Services. Mediterranean Fruit Fly Preventive Release Program CA.gov California Department of Food and Agriculture website2014 [updated 8/25/14; cited 2018]. Available from: <https://www.cdfa.ca.gov/plant/PDEP/prpinfo/>.

66. Tsai RJ, Sievert J, Prado J, Buhl K, Stone DL, Forrester M, et al. Acute illness associated with use of pest strips - seven U.S. States and Canada, 2000-2013. MMWR Morb Mortal Wkly Rep. 2014;63(2):42-3. Epub 2014/01/17. PubMed PMID: 24430101; PubMed Central PMCID: PMCPMC4584652.

**图1。 2010 - 2015年各国使用的有机磷农药平均年吨数**。较暗的阴影表示农药更多的使用。灰色阴影表示在该时间段内没有可用数据。对于2010 - 2015年部分但不是所有年份都有数据的国家，使用了该期间的可用数据。美国数据的来源是[6]; 所有其他国家的数据来源是 [5]。使用mapchart.net创建的地图。

**图S1。 2010 - 2015年按国家/地区每1000平方公里使用的有机磷农药平均年吨数**。较暗的阴影表示每1000平方公里使用更多。灰色阴影表示在该时间段内没有可用数据。对于2010 - 2015年部分但不是所有年份都有数据的国家，使用了该期间的可用数据。美国数据的来源是[6]; 所有其他国家的数据来源是[5]。使用mapchart.net创建的地图。
