# Supplementary material for: Organophosphate exposures during pregnancy and child neurodevelopment: Recommendations for essential policy reforms
Source: PLoS Med. 2018 Oct 24;15(10):e1002671. doi: 10.1371/journal.pmed.1002671 (PMC6200179; doi:10.1371/journal.pmed.1002671)
Supplement: S3 Text — (DOCX) [file pmed.1002671.s004.docx]

**Organophosphate exposures during pregnancy and child neurodevelopment: Recommendations for essential policy reforms**

**Expositions aux pesticides organophosphorés pendant la grossesse et neurodéveloppement de l'enfant : recommandations pour des actions politiques**

**Résumé**

- *Widespread use of organophosphate (OP) pesticides to control insects has resulted in ubiquitous human exposures.*
  - Nous sommes couramment exposés aux pesticides organophosphorés du fait de leur utilisation répandue pour lutter contre les insectes.
- *High exposures to OP pesticides are responsible for poisonings and deaths, particularly in developing countries.*
- L’exposition à de fortes doses de pesticides organophosphorés est responsable d’empoisonnement et de décès notamment dans les pays en voie de développement.
- *Compelling evidence indicates that prenatal exposure at low levels is putting children at risk for cognitive and behavioral deficits and for neurodevelopmental disorders*
- Un faisceau d’indices suggère que des expositions faibles pendant la période prénatale seraient aussi délétères et pourraient être associées à des déficits cognitifs, des troubles du comportement et d’autres troubles du neurodéveloppement chez les enfants.
- *To protect children worldwide, we recommend the following:*
- Pour la protection des enfants dans le monde nous recommandons que :
- *Governments phase out chlorpyrifos and other OP pesticides; monitor watersheds and other sources of human exposures; promote use of integrated pest management (IPM) through incentives and training in agroecology; and implement mandatory surveillance of pesticide-related illness.*
- Les gouvernements interdisent l’utilisation du chlorpyriphos et des autres pesticides organophosphorés ; mettent en place une surveillance des bassins versants et des autres sources d'exposition humaine ; soutiennent la lutte antiparasitaire intégrée par le biais d'incitations et de formations en agroécologie et mettent en œuvre une surveillance obligatoire des maladies liées à ces pesticides.
- *Health professions implement curricula on the hazards from OP pesticides in nursing and medical schools and in continuing medical education courses; and educate their patients and the public about these hazards.*
- La mise en place de programmes de formation sur les dangers liés à l’utilisation des pesticides organophosphorés dans les écoles de soins infirmiers et de médecine ou dans les cours de formation continue des professionnels de santé qui, par la suite, éduqueront leurs patients et le public sur ces dangers.
- *Agricultural entities accelerate the development of non-toxic approaches to pest control through IPM; and ensure the safety of workers through training and provision of protective equipment when toxic chemicals are to be used.*

Les industries agricoles accélèrent le développement d'approches non toxiques pour la lutte antiparasitaire et assurent la sécurité des travailleurs par la formation et la mise à disposition d'équipements de protection lorsque des produits chimiques toxiques sont utilisés.
