# Supplementary material for: Organophosphate exposures during pregnancy and child neurodevelopment: Recommendations for essential policy reforms
Source: PLoS Med. 2018 Oct 24;15(10):e1002671. doi: 10.1371/journal.pmed.1002671 (PMC6200179; doi:10.1371/journal.pmed.1002671)
Supplement: S4 Text — (DOCX) [file pmed.1002671.s005.docx]

Riassunto

• L'uso diffuso di pesticidi organofosfati (OP) contro gli insetti ha comportato l’esposizione ubiquitaria anche nell’uomo.

• Elevate esposizioni ai pesticidi OP sono responsabili di avvelenamenti e morti, in particolare nei

paesi in via di sviluppo.

• Evidenze convincenti indicano che l'esposizione prenatale a livelli bassi di OP rappresenta un fattore di rischio per deficit cognitivi e comportamentali infantili e per disturbi del neurosviluppo.

Per proteggere i bambini in tutto il mondo, raccomandiamo quanto segue:

• In ambito governativo: eliminazione graduale dell’uso di clorpirifos e altri pesticidi OP; monitoraggio dei bacini idrografici e di altre fonti di esposizione umana; promozione della gestione integrata delle infestazioni parassitarie (IPM) utilizzando incentivi e promuovendo la formazione in agroecologia; avvio di una sorveglianza sanitaria obbligatoria per le patologie potenzialmente associate all’esposizione a OP.

• In ambito sanitario: per le professioni sanitarie attuazione di piani di studio e di corsi di formazione continua per medici e infermieri sui rischi derivanti dai pesticidi OP; educazione dei pazienti e della cittadinanza su questi rischi.

• In ambito agricolo: sviluppo di approcci non tossici per il controllo dei parassiti attraverso gestione integrata delle infestazioni parassitarie (IPM); promozione della sicurezza dei lavoratori attraverso la formazione e la fornitura di dispositivi di protezione in caso di utilizzo di prodotti chimici tossici.

.
